# Construction of an immune-related signature for predicting the ischemic events in patients undergoing carotid endarterectomy

**DOI:** 10.3389/fgene.2022.1014264

**Published:** 2022-10-10

**Authors:** Shifu Li, Qian Zhang, Ling Weng, Jian Li

**Affiliations:** ^1^ Department of Neurosurgery, Xiangya Hospital, Central South University, Changsha, China; ^2^ National Clinical Research Center for Geriatric Disorders, Central South University, Changsha, China; ^3^ Department of Neurology, Xiangya Hospital, Central South University, Changsha, China; ^4^ Hydrocephalus Center, Xiangya Hospital, Central South University, Changsha, China

**Keywords:** atherosclerosis, immune-related genes, immune infiltration, ischemic events, molecular subtypes

## Abstract

**Background:** Inflammatory responses have drawn more attention to atherosclerosis; however, the immune-related genes (IRGs) as a prognostic factor in atherosclerotic plaque remain to be fully elucidated. Here, the purpose of this study was to investigate whether the IRGs could be identified as a reliable biomarker for predicting ischemic events in patients undergoing carotid endarterectomy (CEA).

**Methods:** Two datasets GSE97210 and GSE21545 were downloaded from the Gene Expression Omnibus (GEO) database. The dataset GSE97210 was used to explore the significant pathways and differentially expressed IRGs (DEIRGs) between plaques and controls, which were further screened to identify the prognostic DEIRGs in the GSE21545 dataset. The identification of molecular subgroups with the prognostic gene expression patterns was achieved through nonnegative matrix factorization (NMF) clustering. Functional analyses including GO, KEGG, GSVA, and GSEA analyses, and immune analyses including xCell and ssGSEA algorithms were conducted to elucidate the underlying mechanisms. The prognostic risk model was constructed using the LASSO algorithm and multivariate Cox regression analysis.

**Results:** A total of 796 DEIRGs (including 588 upregulated and 208 downregulated) were identified. Nine prognostic DEIRGs were further screened with univariate Cox regression analysis. Two clusters with different prognosis were grouped based on the prognostic DEIRGs. Immune infiltration analysis shows that cluster 2 with a better prognosis presented with a higher immune response than cluster 1. A prognostic model based on seven IRGs (IL2RA, NR4A2, DES, ERAP2, SLPI, RASGRP1, and AGTR2) was developed and verified. Consistent with the immune analysis of the cluster, the immune infiltration in the low-risk group with a better prognosis was also more active than that in the high-risk group. Finally, a nomogram based on the seven genes was constructed, which might have future implications in clinical care.

**Conclusion:** The expression of immune-related genes is correlated with the immune microenvironment of atherosclerotic patients and could be applied to predict the ischemic events in patients undergoing CEA accurately.

## Introduction

Atherosclerosis, a chronic inflammatory disease characterized by the accumulation of lipids, fibrous elements, and calcification within large arteries, results in cardiovascular complications, such as coronary artery disease (CAD) and stroke that remain leading causes of death worldwide (Ross, 1999; Moore and Tabas, 2011). Cerebrovascular events such as transient ischemic attack (TIA) and ischemic strokes (ISs) are closely associated with carotid atherosclerotic plaque rupture or artery stenosis (Brinjikji et al., 2016). Stenosis of the internal carotid artery causes ISs (symptomatic carotid stenosis) in 8%–15% cases due to atherosclerosis (Bonati et al., 2022). Carotid artery stenting (CAS) and carotid endarterectomy (CEA) are the two major surgical operations to restore patency and reduce long-term stroke risks. A recent clinical trial compared their long-term protective effects and found that 5-year non-procedural stroke incidence was 2.5% in each group for fatal or disabling stroke, with no significant difference for any stroke (5.3% with CAS *versus* 4.5% with CEA, *p* = 0.33) (Halliday et al., 2021). These two procedures may not always be beneficial for all patients with carotid atheromatous disease and prevent the occurrence of secondary cardiovascular events. Moreover, the recurrence of ischemic events (including ischemic stroke and myocardial infarction) after CEA is still inevitable (Folkersen et al., 2012). Therefore, identifying patients with elevated risk of ischemic events after undergoing CEA is vital.

Previous studies suggested the causal role of the immune system and inflammation in atherosclerosis (Moore and Tabas, 2011; Tabas and Lichtman, 2017). Several inflammation processes participate in all stages of atherosclerosis and remain a substantial residual cardiovascular risk factor in optimally treated patients. Inflammatory responses provide a series of pathways that link lipids and other traditional risk factors to atherosclerosis instead of supplanting. For example, in the early stages of atherosclerosis, vascular smooth muscle cells (VSMCs) exposed to modified low-density lipoprotein (LDL) accumulated in the subendothelial region and released chemoattractants, including chemokine 2 (CCL2) and CCL5, which promoted the recruitment of monocytes (Quinn et al., 1987; Cushing et al., 1990). Upon entering the intimal region, these monocytes differentiate into macrophages by reacting with macrophage colony-stimulating factor (M-CSF) and other cytokines. The lesional macrophages can engulf “modified” lipoproteins (such as oxidation or aggregation) and generate foam cells, which frequently undergo apoptosis or necrosis to generate a growing “necrotic core” composed of cholesterol esters and cell debris that causes the lesion to rupture. Targeting inflammation by inhibiting leucocyte motility and cytokine release from a range of inflammatory cells is reported to reduce cardiovascular events. A recent meta-analysis demonstrated the efficacy of low-dose colchicine for the secondary prevention of cardiovascular events in patients with coronary artery disease (Fiolet et al., 2021). However, immunotherapies need to be tailored to specific groups of patients with atherosclerotic cardiovascular disease depending on their clinical status. In the LoDoCo2 trial (Nidorf et al., 2020), the incidence of death from noncardiovascular causes was higher in the colchicine group than in the placebo group. Consequently, for precision medicine, it is very important to distinguish patients into different risk groups before treatments, especially according to their immune landscapes.

With the development of high-throughput sequencing and the availability of the large-scale public databases, bioinformatics analyses of gene expression profiles are widely used to identify differentially expressed genes (DEGs), analyze functional pathways, and uncover molecular mechanisms involved in the pathogenesis of atherosclerosis, such as intraplaque hemorrhage, progression, and rupture (Chen et al., 2021; Li et al., 2022). Several studies have been performed by constructing a risk prognostic model to predict the overall survival rates (Huang et al., 2021a; Huang et al., 2021b). However, few studies have answered whether expression patterns from atherosclerosis patients predict future prognosis, such as the occurrence of ischemic events (Folkersen et al., 2012). Previous studies demonstrated that the composition of carotid atherosclerotic plaque (Hellings et al., 2010) and expression levels of genes such as collagenase matrix metalloproteinase-8 (MMP8) (Peeters et al., 2011a) and fatty acid-binding protein 4 (FABP4) (Peeters et al., 2011b) in the carotid atherosclerotic plaque provide prognostic information for future cardiovascular outcomes. However, there are no studies systematically predicting the ischemic events base on the expression profiles and depicting the immune landscapes of patients after undergoing CEA.

In the present study, we first explored the difference in immune-related pathways and characteristics between normal arterial intimae and advanced atherosclerotic plaques. The differentially expressed immune-related genes (DEIRGs) were identified between plaque and control samples, and the genes that demonstrated prognostic ability by using univariate Cox regression analysis were further screened. Based on the hub prognostic immune-related genes (IRGs), patients with atherosclerotic plaques were divided into two clusters by using the Non-negative Matrix Factorization (NMF) algorithm. The survival analysis, immune characteristics, and functional processes between the two clusters were reconnoitered. Finally, a prognostic model based on IRGs was constructed and validated, and the patients were classified into low and high-risk groups who presented with different event-free survival during follow-up. This study hypothesizes that the IRGs could predict the ischemic events in patients endergoing CEA from the perspective of prognostic effects.

## Methods and materials

### Datasets and preprocessing

**FIGURE 1:**
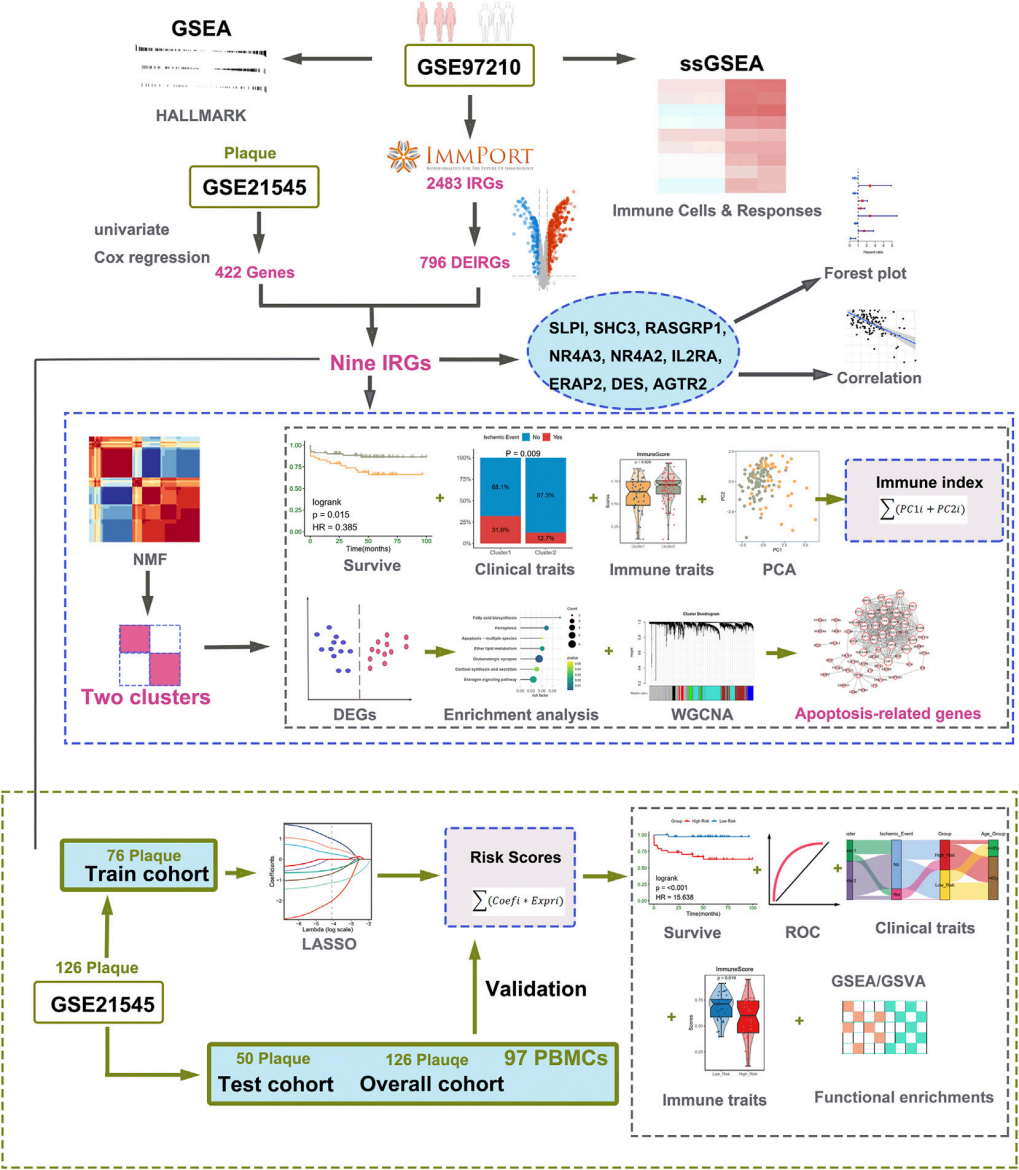
Workflow of data analysis in our present work. DEGs, differentially expressed genes; IRGs, immune-related genes; DEIRGs, differentially expressed immune-related genes; GSEA, gene set enrichment analysis; ssGSEA, single sample gene set enrichment analysis; GSVA, gene set variation analysis; NMF, Non-negative Matrix Factorization; PCA, principal component analysis; ROC, receiver operating characteristic curve; GO, Gene Ontology; KEGG, Kyoto Encyclopedia of Genes and Genomes; LASSO, least absolute shrinkage, and selection operator; WGCNA, weighted gene co-expression network analysis.

Microarray data, containing two transcription profiles (GSE97210 and GSE21545) were obtained from the NCBI GEO database (https://www.ncbi.nlm.nih.gov/geo/). The list of IRGs was collected from the immunology database and analysis portal (ImmPort) (Bhattacharya et al., 2014). In our research, we choose two datasets: one includes the expression profile of atherosclerotic plaques and normal arterial tissues, and the other includes the expression profile of atherosclerotic plaques and prognostic information of patients undergoing CEA. The dataset of GSE97210 containing six human samples (three normal arterial intimae and three advanced atherosclerotic plaques) was conducted at the platform of GPL16956 (Bai et al., 2019). The GSE21545 dataset was based on an Affymetrix^®^ platform (GPL570) and included 126 plaques and 97 peripheral blood mononuclear cells (PBMCs) samples. The gene expression profiles and clinical data of GSE21545 were downloaded from GEO (Folkersen et al., 2012). The clinical features and descriptions of the GSE21545 dataset are presented in Table 1. Of the ischemic events, seven were myocardial infarctions and 18 were ischemic strokes (Folkersen et al., 2012). If multiple probes were matched with one gene, the probe with maximal median values of expression was annotated into the homologous gene symbol on the basis of the platform’s annotation information. Our study design is briefly described in the flow chart (Figure 1).

**TABLE 1:** Detailed information of GSE21545 datasets.

Samples		Plaque (126)			PBMCs (97)
		Training Cohort (76)	Test Cohort (50)	Overall Cohort (126)	
Status	Ischemic	14 (18.42%)	11 (22.00%)	25 (19.84%)	21 (21.65%)
	No-Ischemic	62 (81.58%)	39 (78.00%)	101 (80.15%)	76 (78.35%)
Age	≤ 65	21 (27.63%)	14 (28.57%)	35 (28.00%)	27 (71.88%)
	>65	55 (72.37%)	35 (71.43%)	90 (72.00%)	69 (28.12%

PBMCs, peripheral blood mononuclear cells.

### Immune landscapes between atherosclerotic plaques and normal arterial tissues

The gene sets of hallmarks were obtained from the Molecular Signatures Database (MSigDB) (Liberzon et al., 2015), and the Z-score of hallmarks was quantified using a gene set variation analysis (GSVA) algorithm (R package “gsva”) based on transcriptomic profiling data of GSE97210 dataset. The “fgsea” package in R was used to display the enrichment results of GSEA. We also adopted the ssGSEA algorithm to assess the 28 immune infiltrating cells and 17 immune responses between the two groups. The results are displayed in a heat map using “ComplexHeatmap” packages. The significantly different immune cell and responses were compared using “Wilcoxon test”.

### Identification of differentially expressed and prognostic IRGs in atherosclerotic plaques

The expression profiles of 2483 IRGs from ImmPort were first extracted in the dataset GSE97210.

Differential expression analysis was performed using the “limma” package in R software to identify differentially expressed IRGs (DEIRGs) between the plaques and control groups. The DEGs were screened with the criteria of |log2FoldChange| > 1 and *p* < 0.05. Then, a univariate Cox regression analysis was carried out to identify prognosis-related genes of patients undergoing CEA from dataset GSE21545. We overlapped the DEIRGs and genes with a log-rank *p* < 0.05 to identify the hub IRGs.

### Correlation among hub IRGs and classification of molecular subtypes

To uncover the association among genes, the expressive correlation among them was calculated using Pearson’s method, and the results were presented using “corrplot” and “ggraph” packages. NMF is a dimensionality reduction approach for learning a parts-based and linear representation of non-negative data. NMF clustering was applied for the identification of new clusters using the “nmf” R package based on the hub IRGs. Kaplan-Meier (KM) curve shows the relationship between two clusters and clinical outcome, and the log-rank test was used to evaluate differences using ‘survival’ and “survminer” packages.

### Biological functions and immune characteristics between two subtypes

The DEGs between two clusters were identified using the “limma” package with the criteria of |log2FoldChange| > 0.5 and *p* < 0.05. The “clusterProfiler” package was used to enrich the biological processes (BP) of Gene Ontology and Kyoto Encyclopedia of Genes and Genomes (KEGG) pathways of DEGs.

xCell (Aran et al., 2017), a novel gene signature-based method, was used to infer 64 immune and stromal cell types and to estimate the immune scores and stromal scores. The abundance of immune infiltrating cells and immune response were also evaluated by using the aforementioned ssGSEA method. We also compared the expression levels of 11 checkpoint-related genes (PDCD1, CD274, CTLA4, ICOS, HAVCR2, CD80, CD47, BTLA, TIGIT, SIRPA, and VTCN1) between the two clusters. The relationships between nine hub IRGs and immune characteristics were calculated using Pearson’s method and visualized by using “ggplot2” package.

Principal component analysis (PCA) was conducted based on nine hub IRGs by using the prcomp function to assess the distinguishable ability for identified subtypes. PC1 and PC2 were extracted to form signature scores. Later, we applied a method similar to GGI to construct the immune index (Sotiriou et al., 2006).

Immune Index=∑(PC1i+PC2i)

where i shows the expression of prognostic DEIRGs.

The relationships between the immune index and immune characteristics were also assessed.

### Identification of the correlation of nine IRGs and apoptosis-related genes

From the above hallmark enrichment analysis between plaques and normal tissue, we observed that the apoptosis process was also involved in this pathogenesis. Therefore, we identified the apoptosis-related genes that are correlated with the nine hub IRGs. Three methods were used to screen for apoptosis-related genes.

1) Pearson correlation analysis was performed to analyze the relevance between genes and nine hub IRGs in GSE21545. IRGs-related genes were defined as genes significantly related to at least one hub IRGs (|Pearson’s correlation coefficient | ≥ 0.5 and *p* < 0.05).
2) We evaluated the top 5,000 variant genes to test their availability and used the R package termed “WGCNA” to construct a gene co-expression network. The power values corresponding to an independent index of R2 = 0.85 were selected. The minimum number of genes in each module was 30, and the threshold of merging modules was set to 0.25 using the dynamic cutting tree method. The module with most correlated with the “APOPTOSIS” trait calculated by GSVA method with significance (*p* < 0.05) was assumed to be the key module for further analysis by using the moduleTraitCor and moduleTraitPvalue algorithms.
3) The list of apoptosis-related genes was extracted from MSigDB. The search strategy included the following keywords: “apoptosis” and “*Homo sapiens*.” A total of 2747 apoptosis-related genes were listed.

By integrating the above methods, the final apoptosis-related genes correlated with hub IRGs were identified. The protein-protein interaction (PPI) network was constructed using the Search Tool for the Retrieval of Interacting Genes database (STRING, www.string-db.org) with medium confidence of 0.4 and further visualized using Cytoscape software (v3.8.2). The biological functions of these apoptosis-related genes were identified using an online tool Metascape (Zhou et al., 2019).

### Establishment and verification of a prognostic model based on IRGs

A total of 126 patients in GSE21545 were divided into a training cohort and a test cohort at a ratio of 6:4. The least absolute shrinkage and selection operator (LASSO) Cox regression was used to identify the hub IRGs by using the “glmnet” package. We chose the minimum lambda as the optimal value. The genes used for the establishment of the risk model were determined by multivariate Cox regression analysis. The risk score for each sample was calculated as follows: Risk scores = ∑(coefficienti * expression of signature genei). After calculating the risk scores, patients were categorized into two groups. Patients with risk scores more than the medium value were grouped into the high-risk group and others into the low-risk group. The prognostic evaluation ability of the risk scores was evaluated by plotting the KM survival curve and receiver operating characteristic (ROC) curve using “timeROC” package. The test cohort, overall cohort, and 97 PBMC samples in GSE21545 were used to validate the prognostic model.

### Biological functions and immune characteristics between the two risk groups in the training cohort

GSVA and GSEA method were used to identify the different hallmark and KEGG pathways, respectively. xCell algorithm was applied to assess the stromal and immune scores between the high- and low-risk groups. As before, we utilized the ssGSEA method to assess the abundance of different immune cells and responses.

### Construction of nomogram

To improve the prognostic risk stratification of patients undergoing CEA and assist in clinical diagnosis and treatment, we constructed a nomogram model with the selected genes. It was used as a quantitative tool to predict the prognosis of patients after CEA. The effectiveness of the model was evaluated using a calibration curve.

### Statistics analysis

All statistical analyses were performed using R software (version R-4.1.0). The Wilcoxon test was used for statistical analysis between the two groups. The KM curve plotted the relationship between score and clinical outcome, and the log-rank test was used to evaluate differences using “survival” and “survminer” packages. Univariate and multivariate Cox regressions were performed with the “survival” package. The relationships of genes with genes and genes with immune cells were constructed by using Pearson’s correlation method. LASSO regression analysis was carried out using the “glmnet” package. Unsupervised cluster analysis was performed using the R package “NMF”. *p* < 0.05 was considered to indicate statistical significance. The significance level is denoted as follows: *p < 0.05, **p < 0.01, and ***p < 0.001.

## Results

### Depicted biological pathways, the immune landscapes, and DEIRGs between plaques and controls

**FIGURE 2:**
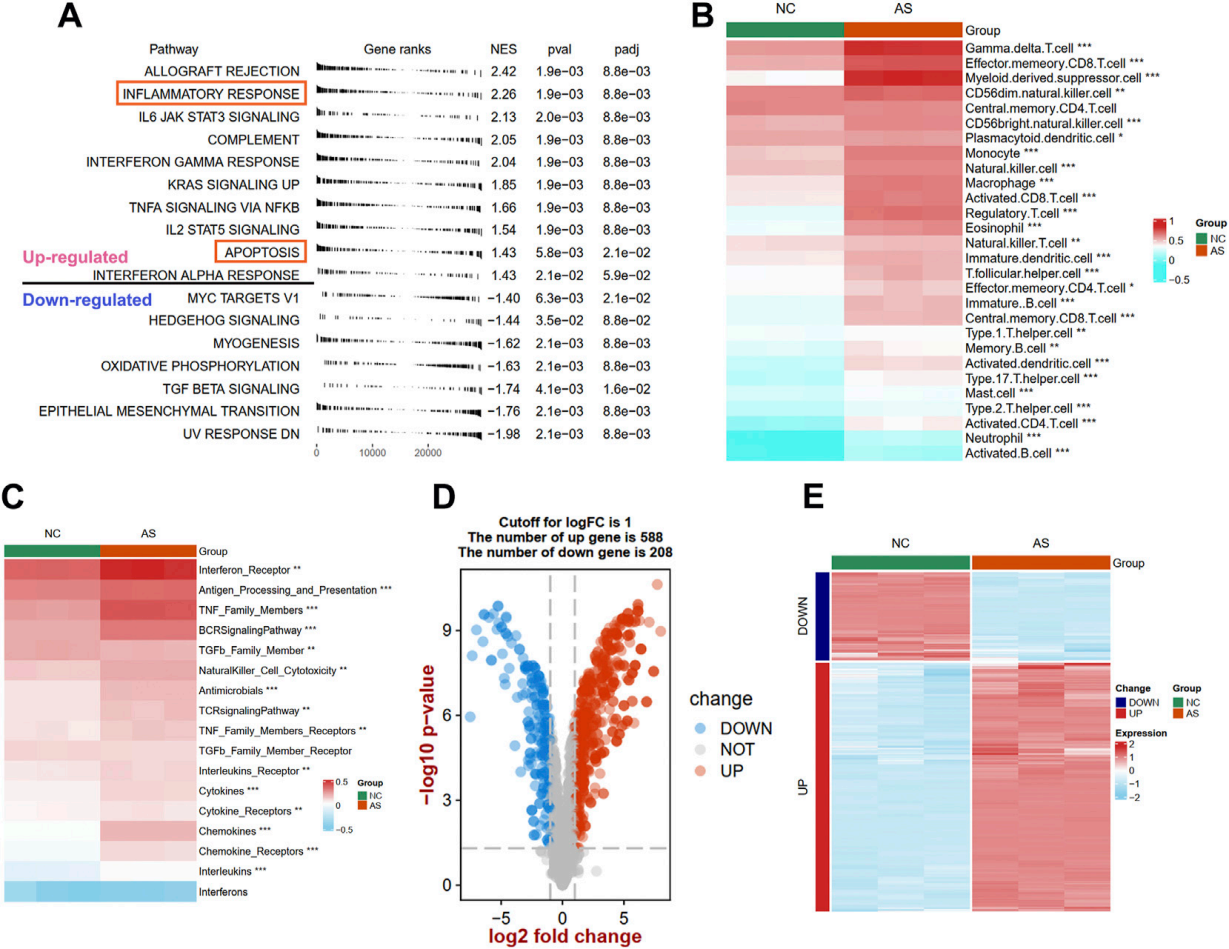
Identification of different function pathways, immune infiltration, and DEIRGs in plaque compared with the normal arterial tissues. **(A)** Up and downregulated hallmark pathways between plaque and controls in GSEA analysis. The difference of immune cell **(B)** and immune responses **(C)** between plaque and controls in ssGSEA analysis. A volcano plot **(D)** and heat map **(E)** shows the differentially expressed immune-related genes (DEIRGs) between plaque and controls. Significance level was denoted by *p value < 0.05, **p value < 0.01, ***p value < 0.001.

To uncover the different biological pathways between plaques and controls, a GSEA method was used. In our GSEA results, we observed that the immune-related hallmark pathways (such as inflammatory response, IL6/JAK/STAT3 signaling, and complement pathways) were up-regulated in atherosclerotic plaque samples compared with normal tissues in the GSE97210 dataset (Figure 2A). The apoptosis process was also identified in atherosclerotic plaque samples. To further understand the immune infiltration in plaques, we applied an ssGSEA method to uncover 28 differential immune cells and 17 differential responses between plaques and controls. As shown in Figure 2B; Supplementary Figure S1A, plaques had more immune cell infiltration than the controls, such as macrophages, activated CD8 T cells, and mast cells. The heat map depicts the differences in immune responses between plaques and controls, showing that interferon receptors, antigen processing and presentation, and TNF family number were more active in plaque tissues (Figure 2C; Supplementary Figure S1B).

To explore the expression patterns of IRGs between plaques and controls, a total of 1926 IRGs were detected in the GSE97210 dataset, and their expression profiles were extracted to identify the DEIRGs. We identified 796 DEIRGs (including 588 upregulated and 208 downregulated), and the results are shown in a volcano plot (Figure 2D) and a heat map (Figure 2E).

### Molecular subgroups clustered by prognostic DEIRGs

**FIGURE 3:**
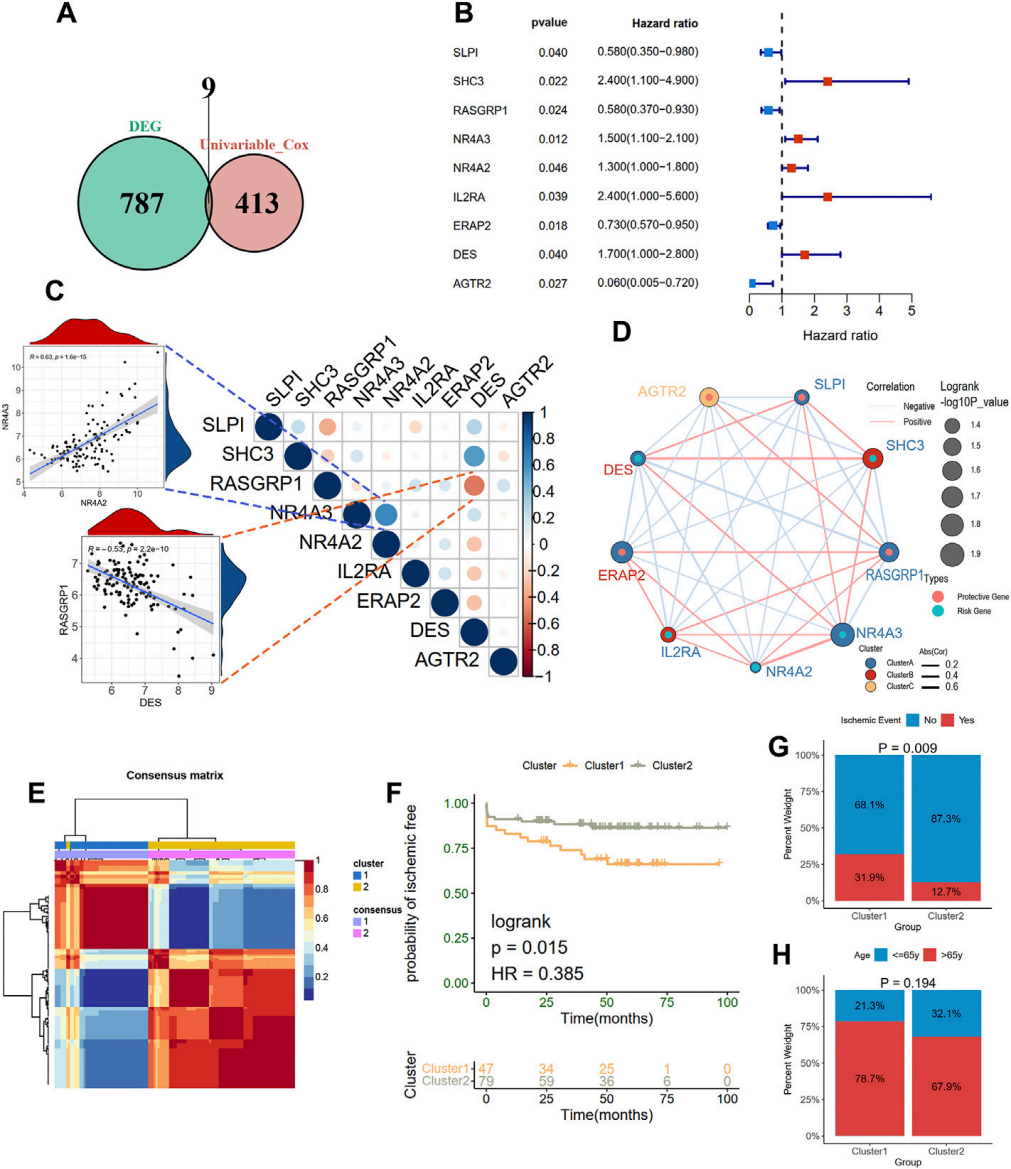
Clustering two molecular subgroups through nonnegative matrix factorization (NMF) method. **(A)** Screening nine prognostic DEIRGs by overlapping the DEIRGs and prognostic genes with univariate Cox regression analysis. **(B)** A forest plot showing the hazard ratio of nine prognostic DEIRGs. **(C)** and **(D)** indicating the expression correlations of the nine prognostic DEIRGs. The enlarged panels show the most positive and negative correlation. **(E)** Identification of two clusters with the optimal value for consensus clustering. **(F)** Survive analysis of two distinct clusters. A histogram shows the proportion of the occurrence of an ischemic event **(G)** and age group **(H)** in two clusters.

To elucidate the relationship between immune-related molecular patterns and prognosis in atherosclerotic patients, we first found the prognostic DEIRGs and then clustered the samples into two patterns based on the expression of them using NMF method. A total of 422 prognosis-related genes associated with event-free survival (Supplementary Table S1) were identified through univariate Cox regression analysis in the GSE21545 dataset. By intersecting the 422 genes with the above DEIRGs, nine prognostic DEIRGs were overlapped (Figure 3A; Table 2). The forest plot shows that SLPI, RASGRP1, ERAP2, and AGTR2 may serve as protective genes, and SHC3, NR4A3, NR4A2, IL2RA, and DES may act as risk genes (Figure 3B). To explore the expression association between the nine different prognostic DEIRGs, we depicted the correlation patterns between them (Figures 3C,D). According to the expression patterns of the nine genes, they were classified into three clusters by hierarchical clustering (Supplementary Figure S2A). The enlarged panels show the most positive and negative expression correlations between them; NR4A3 and NR4A2 were most positively correlated (R = 0.63, *p* < 0.001), and RASGRP1 and DES were most negatively correlated (R = −0.53, *p* < 0.001). Based on the nine prognostic DEIRGs, the 126 patients in the GSE21545 dataset were divided into two clusters by applying the NMF algorithm (Figure 3E; Supplementary Figure S2B). We compared the ischemic events-free survival of the two clusters and found that cluster two had a better prognosis (HR = 0.385, *p* = 0.015, Figure 3F). The histogram of the frequency distribution revealed that cluster 1 had a larger proportion of ischemic events than cluster 2 (Chi-square test, *p* = 0.009) (Figure 3G), while there was no difference in age distribution between the two clusters (Chi-square test, *p* = 0.194) (Figure 3H).

**TABLE 2:** Gene descriptions and univariate Cox regression of nine prognostic immune-related genes.

Gene name	Gene description	Chromosome	Gene type	HR (95%CI)	*p* Value
SLPI	Secretory leukocyte peptidase inhibitor	20	protein_coding	0.58 (0.35–0.98)	0.0399
SHC3	SHC adaptor protein 3	9	protein_coding	2.4 (1.1–4.9)	0.0219
RASGRP1	RAS guanyl releasing protein 1	15	protein_coding	0.58 (0.37–0.93)	0.0245
NR4A3	Nuclear receptor subfamily 4 group A member 3	9	protein_coding	1.5 (1.1–2.1)	0.0122
NR4A2	Nuclear receptor subfamily 4 group A member 2	2	protein_coding	1.3 (1–1.8)	0.0459
IL2RA	Interleukin 2 receptor subunit alpha	10	protein_coding	2.4 (1–5.6)	0.0388
ERAP2	Endoplasmic reticulum aminopeptidase 2	5	protein_coding	0.73 (0.57–0.95)	0.0175
DES	Desmin	2	protein_coding	1.7 (1–2.8)	0.04
AGTR2	Angiotensin II receptor type 2	X	protein_coding	0.06 (0.0051–0.72)	0.0266

CI, confidence interval.

### Biological functions and immune characteristics between the two subtypes

We further explore the different biological function and immune characteristics between two immune-related subtypes to find the potential biological difference causing the prognostic difference between two groups. A total of 277 DEGs were identified between the two clusters (Supplementary Figure S3A), and the expression patterns are depicted with a heat map (Supplementary Figure S3B). Results of BP analysis showed that these DEGs were enriched in positive regulation of cell adhesion, T cell differentiation, and leukocyte cell-cell adhesion (Supplementary Figure S3C). KEGG enrichment analysis showed that the DEGs were enriched in fatty acid biosynthesis, ferroptosis, and apoptosis pathways (Supplementary Figure S3C).

**FIGURE 4:**
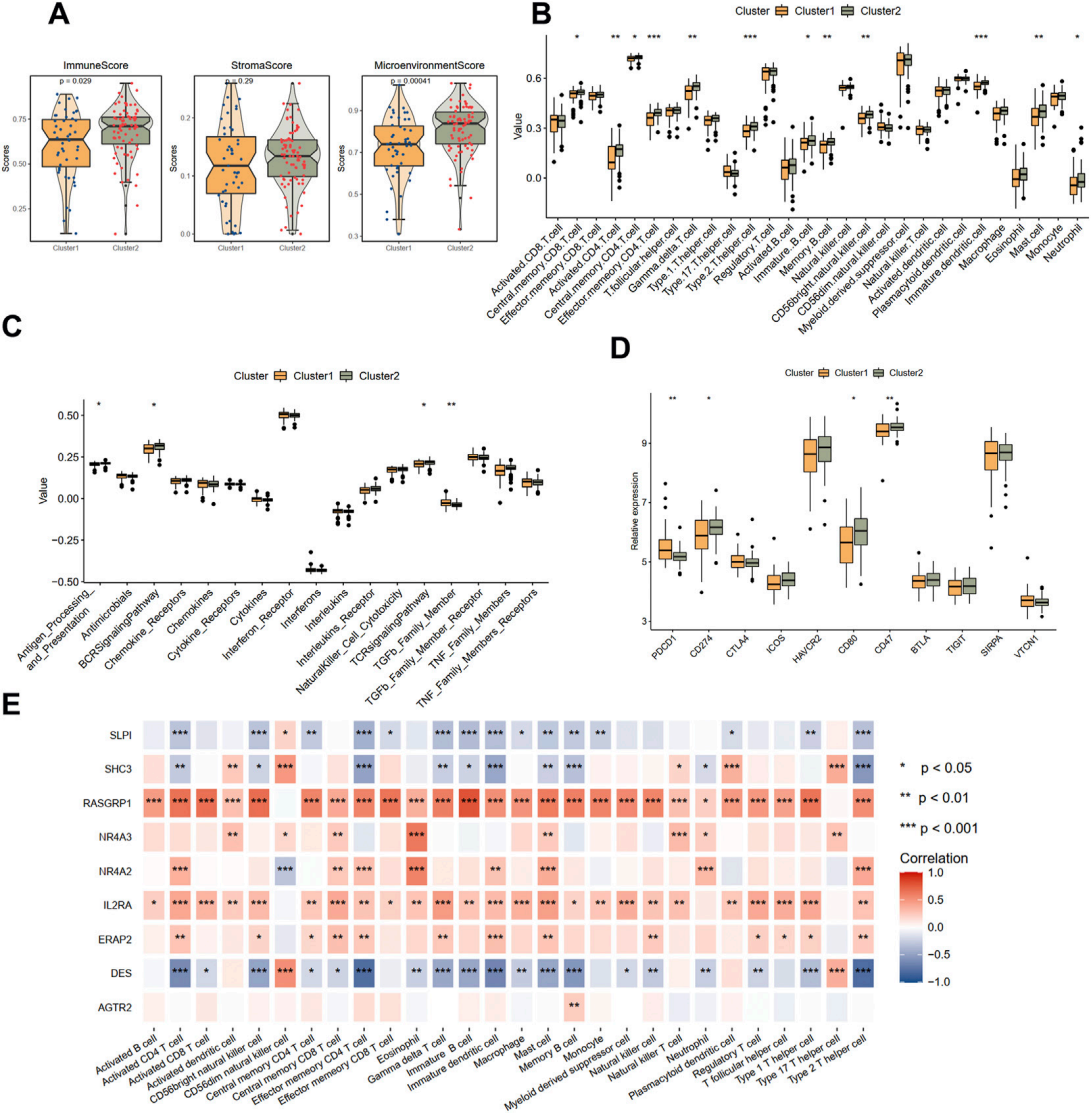
Immune landscapes between two different immune-related clusters. **(A)** The comparison of the immune score, stromal score, and microenvironment score in two clusters with xCell analysis. **(B)** The comparison of 28 immune cells in two clusters with ssGSEA analysis. **(C)** The comparison of 17 immune responses in two clusters with ssGSEA analysis. **(D)** The expression levels of immune checkpoint genes in two clusters. **(E)** The correlations between immune cells and nine immune-related genes. Significance level was denoted by *p value < 0.05, **p value < 0.01, ***p value < 0.001.

Then, the immune condition of the plaque tissues of each patient was evaluated using the xCell algorithm and compared between the two clusters. Surprisingly, as shown in Figure 4A, the plaque tissues in cluster 2 showed a higher immune score and microenvironment score, while the stromal score did not differ. We further explored the immune characteristics, including 28 immune cells, 17 immune responses, and expression levels of 11 immune checkpoint genes. Consistent with the xCell analysis, we observed that cluster 2 had more immune cell infiltration and more active immune responses. Specifically, the abundances of central memory CD8 T cell, activated CD4 T cell, gamma delta T cell, memory B cell, mast cell, and neutrophil were elevated in cluster 2 (Figure 4B). From the perspective of immune responses, the antigen processing and presentation, BCR signaling pathway, and TCR signaling pathway significantly were more active in cluster 2, while the activity of the TGFβ family member was suppressed (Figure 4C). The expression levels CD274, CD80, and CD47 were significantly up-regulated in cluster 2, whereas PDCD1 was significantly down-regulated, compared with cluster 1 (Figure 4D).

The correlations between nine hub DEIRGs with immune characteristics were further identified (Figure 4E; Supplementary Figures S4A,S4B). We observed that the genes were strongly correlated with most immune characteristics, except for AGTR2, which only significantly interplayed with memory B cells. Among these prognostic genes, RASGRP1, identified as a protective gene above, was almost positively related to all immune characteristics. Simultaneously, DES, a risk gene, was found negatively correlated with most immune characteristics. However, another protective gene, SLPI, was also found negatively correlated with most immune characteristics. These results suggested that cluster 1 is immunosuppressive while cluster 2 is relatively immune-activated and that clustering based on DEIRGs is closely correlated with prognosis and the immune microenvironment in plaques.

### Generation of immune index

**FIGURE 5:**
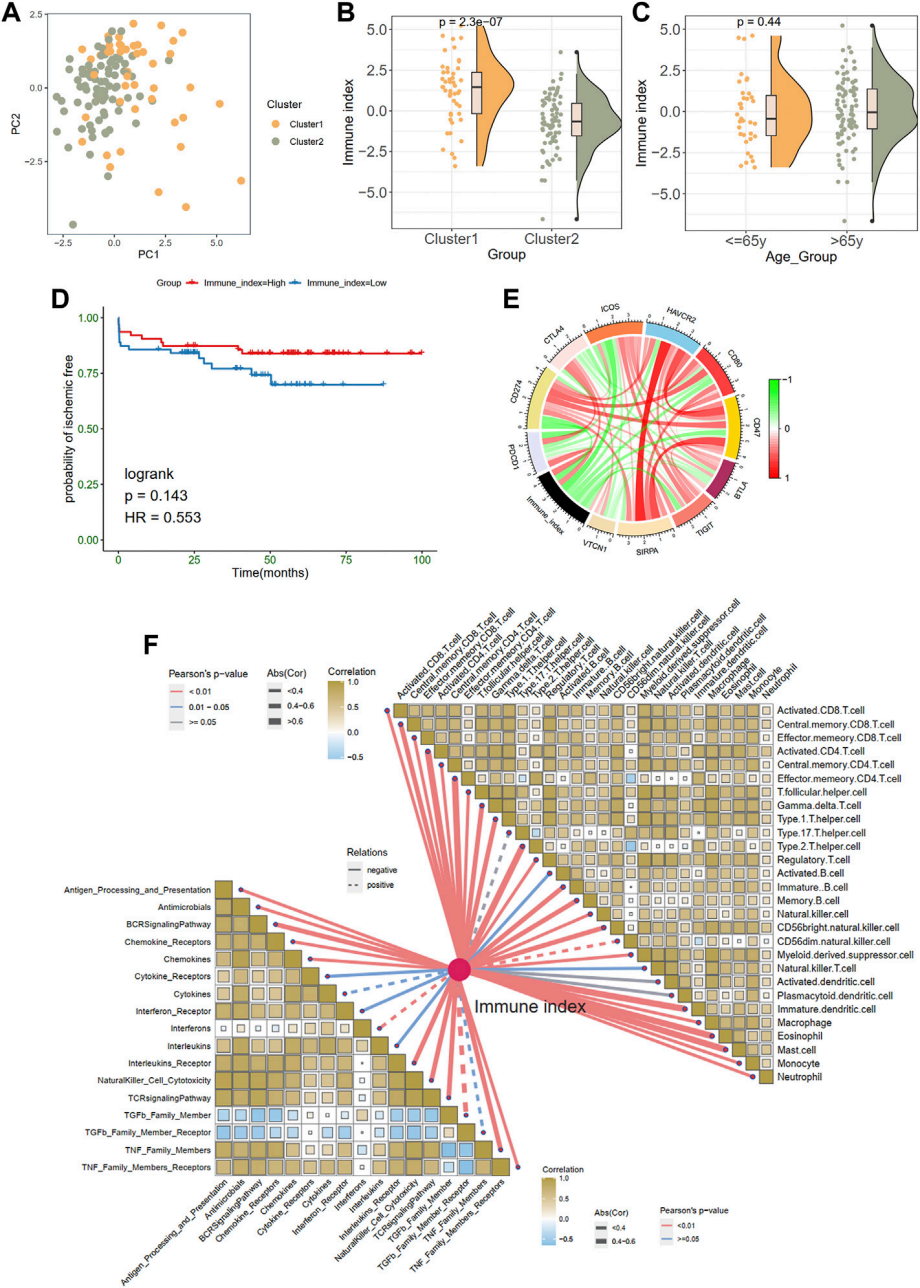
Generation of immune index. **(A)** Principal component analysis. **(B)** The comparison of an immune index between two clusters. **(C)** The comparison of an immune index between different age groups. **(D)** Survive analysis of high- and low-immune index. **(E)** The relationships between the immune index and immune checkpoint genes. Green indicates the negative relations and red represents the positive relations. **(F)** The relationships between the immune index with the immune cells and responses.

Considering the unique heterogeneity of immune patterns, we defined an indicator called immune index to establish a scoring system to comprehensively quantify the immune pattern of patients with plaques. On the basis of nine prognostic DEIRGs, PCA plot shows the distributions of ech samples (Figure 5A). Further analysis revealed a higher immune index in cluster 1 than in cluster 2 (Figure 5B), whereas no difference was observed between different age groups (≤65 years old vs. > 65 years old; Figure 5C). However, no significant prognostic difference was observed in the high- and low-immune index group in KM survival analysis (Figure 5D).

In addition, we explored the associations between immune index and immune characteristics. The immune index was negatively related to most immune checkpoint genes in the chord diagram (Figure 5E). Furthermore, there were significant adverse correlations between the immune index and immune infiltration cells and immune responses (Figure 5F). Specifically, monocytes eosinophils, effector memory CD8 T cells, and central memory CD4 T cells were most negatively correlated with immune index (abs (Cor) > 0.6).

### Identification of the correlation between nine IRGs and apoptosis-related genes

**FIGURE 6:**
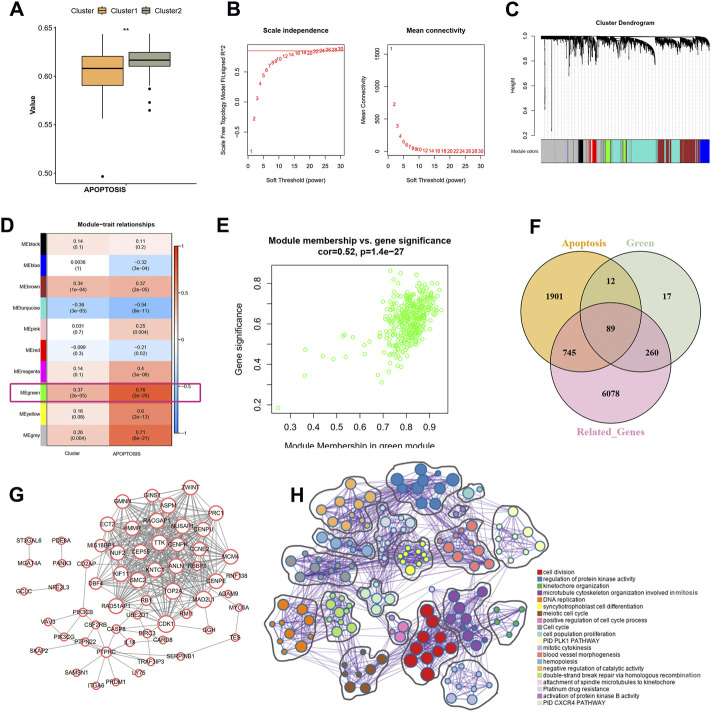
Identification of the correlation of apoptosis-related genes and nine IRGs. **(A)** The difference scores of apoptosis process between two clusters with GSVA analysis. **(B)** Correlation between soft threshold power and scale-free topology model with WGCNA analysis. **(C)** Cluster tree of coexpression modules with WGCNA analysis. Different colors represent different modules. **(D)** The module-trait relationships with WGCNA analysis. **(E)** Screening the green module as the key module. **(F)** Intersecting the apoptosis-related genes correlated with nine IRGs. Apoptosis indicates the apoptosis-related genes in MSigDB database. Green means the green module genes in WGCNA analysis. Related-genes mean those genes correlated with nine IRGs in GSE21545 dataset. **(G)** Protein-protein network of apoptosis-related genes. **(H)** The biological function of apoptosis-related genes. Different colors indicate different pathways.

Based on the GSVA results of the hallmark pathways, we observed a significant difference of apoptosis pathway between the two clusters (Figure 6A). Therefore, we hypothesized that the apoptosis process may also be involved in the ischemic events atherosclerotic plaque. To identify the correlation between hub IRGs and ARGs, we first used a WGCNA algorithm to find the co-expression modules that are high correlated with apoptosis pathway. The top 5,000 genes with median absolute deviation in 126 plaques of the GSE21545 dataset were selected to construct the co-expression network. With the absolute value of the correlation coefficient >0.85, we chose 24 as the optimal soft threshold for constructing scale-free networks (Figure 6B). The 5,000 genes were clustered into 10 modules based on hierarchical clustering under optimal soft-thresholding power (Figure 6C). Then, the correlations between modules with clinical traits were established (Figure 6D). The result revealed that the green module was strongest related to the different immune clusters (cluster 1 and cluster 2) and the “APOPTOSIS” trait calculated by the GSVA method (Figures 6D,E). Secondly, a list of 7172 genes that are at least correlated with the nine prognostic DEIRGs was identified, and the relationship among genes is displayed in Supplementary Table S2. Last, a total of 2474 ARGs were extracted from MSigDB. After intersecting the genes from the above three methods, 89 ARGs correlated with hub IRGs were identified (Figure 6F; Supplementary Table S3). The PPI network was constructed in the STRING database (Supplementay Figure S5), and the genes that interacted with others were further visualized in Cytoscape software (Figure 6G). Through the Metascape tool, the functions of the 89 genes were enriched, and the results showed that these genes are involved in cell division, regulation of protein kinase activity, and DNA replication, all correlated with the apoptosis process.

### Construction of the prognostic model based on prognostic IRGs

**FIGURE 7:**
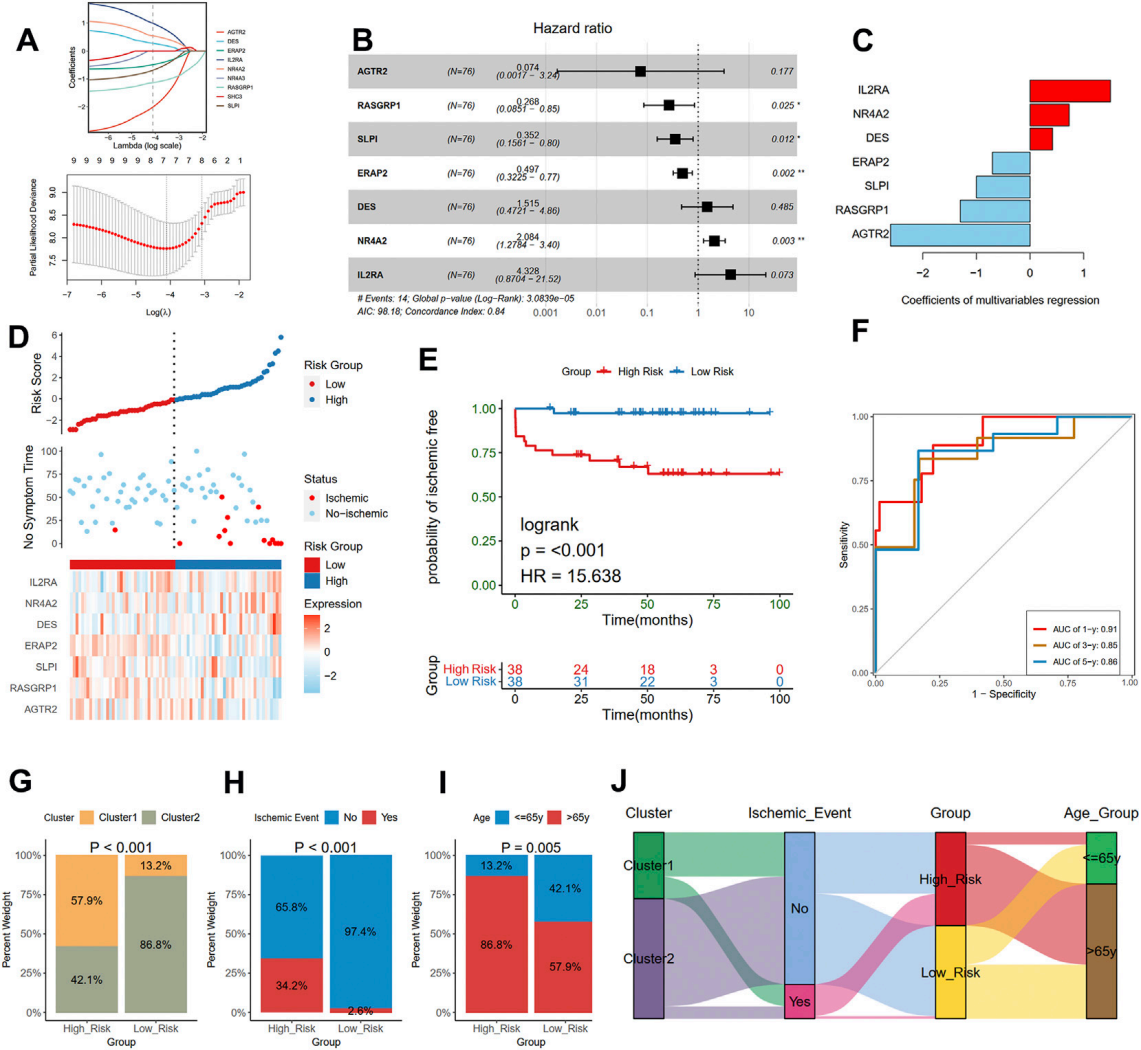
Construction of a risk model in the training cohort. **(A)** Feature selection by LASSO regression (down) and the coefficients change of different genes with different lambda (up). **(B)** Multivariate Cox regression depicted with a forest plot. **(C)** The coefficients of selected seven genes in multivariate Cox regression. **(D)** Distribution of risk score (up) and ischemic status (middle) of atherosclerotic patients in the high and low-risk groups, and heat map (down) illustrating the expression patterns of the seven model genes in the two groups. **(E)** Survival curve of the atherosclerotic patients in the two groups. **(F)** Time-dependent ROC curve of the risk model. A histogram shows the relative proportion of different clusters **(G)**, ischemic event **(H)**, and age group **(I)** in two risk groups. **(J)** A Sankey diagram showing the distribution of different groups.

The risk score was calculated to assess ischemic risk in the individual patients. The 126 plaque samples were randomly assigned to the training cohort (76 samples) and the test cohort (50 samples). LASSO analysis identified seven IRGs from the nine prognostic IRGs as candidate prognostic factors in the training dataset (Figure 7A). Then, a multivariate Cox regression model was constructed based on the seven IRGs and is presented with a forest plot (Figure 7B). In accordance with the constructed prognostic model, each patient was assigned with a risk score as follows: Risk score = (1.465 * IL2RA) + (0.734 * NR4A2) + (0.415 * DES) + (−0.699 * ERAP2) + (−1.043 * SLPI) + (−1.315 * RASGRP1) + (−2.607 * AGTR2). The coefficients of the seven genes are listed in Figure 7C. The training cohorts were separated into low-risk and high-risk groups depending on the median value of the score (Figure 7D). The KM curves showed survival differences between the two groups. Patients in the low-risk group had a better prognosis in the training cohort (log-rank test, < 0.001; Figure 7E). Time-dependent ROC analysis was applied to further evaluate the prediction efficiency of the risk scores, with the areas under the curve (AUCs) of 1, 3, and 5 years being 0.91, 0.85, and 0.86, respectively, in the training cohort (Figure 7F).

Furthermore, we compared the different clinical traits of different risk groups. We observed that the high-risk group had a larger proportion of cluster 1 (Chi-square test, *p* < 0.001; Figure 7G), which presented a poor prognosis as mentioned before. We also found that the proportion of the occurrence of ischemic events in the high-risk group was 34.2%, significantly higher than that in the low-risk group (Chi-square test, *p* < 0.001; Figure 7H). Moreover, the high-risk group had a larger proportion of old patients than the low-risk group (86.8% vs. 57.9%; Chi-square test, *p* = 0.005; Figure 7I). The relationships among different groups were further visualized with a Sankey diagram (Figure 7J).

### Different biological pathways and immune infiltration between high- and low-risk groups

**FIGURE 8:**
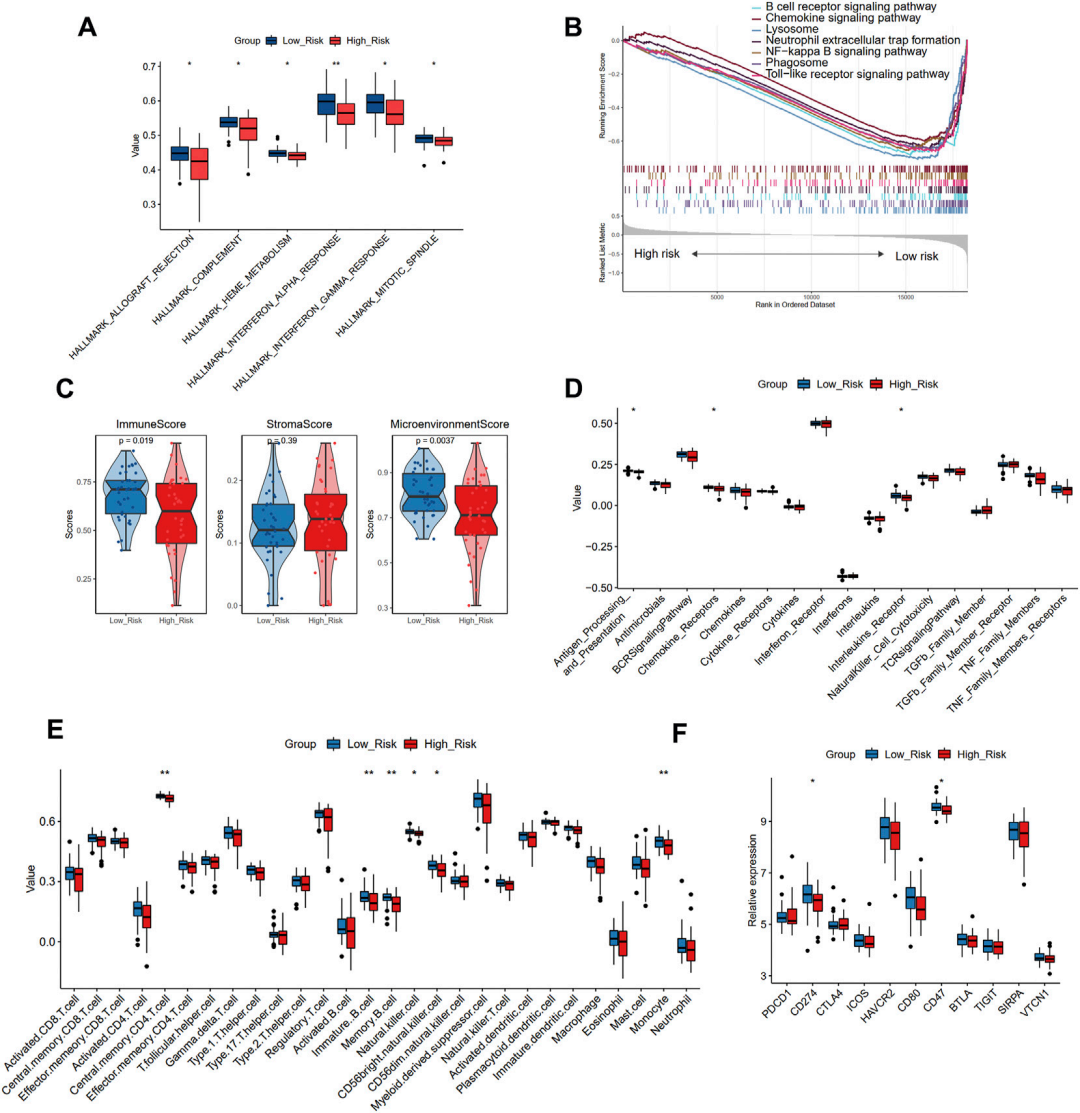
The different biological pathways and immune microenvironment between high- and low-risk groups. **(A)** The significant different hallmark pathways in two groups with GSVA analysis. **(B)** The enrichment of KEGG pathways in two groups with GSEA analysis. **(C)** The comparison of the immune score, stromal score, and microenvironment score in two groups with xCell analysis. **(D)** The comparison of 17 immune responses in two groups with ssGSEA analysis. **(E)**The comparison of 28 immune cells in two groups with ssGSEA analysis. **(F)** The expression levels of immune checkpoint genes in two risk groups. Significance level was denoted by *p value < 0.05, **p value < 0.01, ***p value < 0.001.

To further uncover the different biological mechanisms between high- and low-risk groups in the training cohort, we applied GSVA and GSEA algorithms to enrich the significant hallmark and KEGG pathways, respectively. GSVA showed that the high-risk group had significantly lower scores in six hallmark pathways, including allograft rejection, complement, interferon-alpha and gamma response, and mitotic spindle (Figure 8A). GSEA also demonstrated that compared with the high-risk group, the low-risk group was more enriched in the B cell receptor signaling pathway, chemokine signaling pathway, and lysosome (Figure 8B). Thus, the low-risk group seems to present an immune-activated status.

To verify this finding, we further applied xCell and ssGSEA to assess the immune microenvironment of different risk groups. The high-risk group showed a smaller immune score and microenvironment score than the low-risk group, while no significant difference was observed in the stromal score, further suggesting that the high-risk group may have suppressed immune function (Figure 8C). Immune responses showed decreasing trends from the low-risk group to the high-risk group (Figure 8D), and the antigen processing and presentation, chemokine receptors, and interleukin receptor activities significantly differed between the two groups. In addition, the decreasing trends were also observed in immune cell infiltration (Figure 8E), indicating that the low-risk group had a larger abundance of central memory CD4 T cells, immature B cells, memory B cells, natural killer cells, CD56dim natural killer cells, and monocytes than the high-risk group. We further compared the expression levels of 11 immune checkpoint genes and found that CD274 and CD47 were significantly up-regulated in the low-risk group than the high-risk group (Figure 8F). Combined with the above results, it may be deduced that the immune cell infiltration in patients with atherosclerotic plaque may serve as a protective prognosis factor, consistent with the results of our cluster analysis.

### Validation of the constructed prognostic model

**FIGURE 9:**
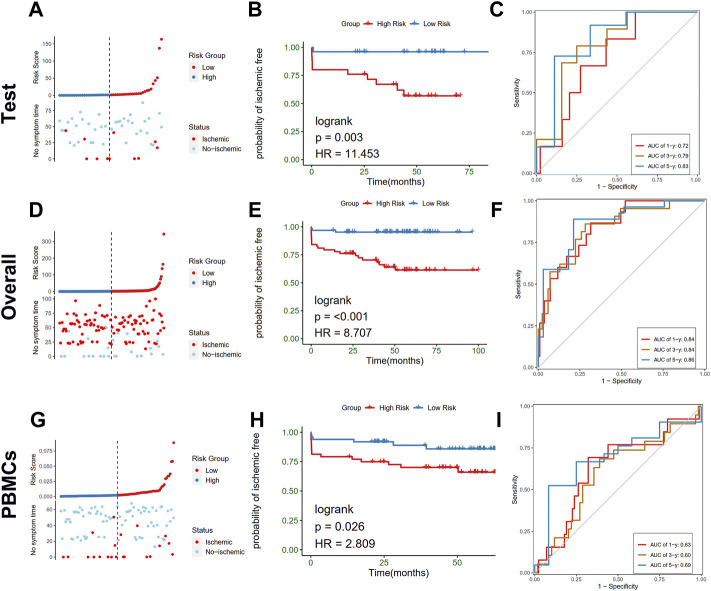
Validation of the risk model in test, overall, and PBMCs cohort. **(A–C)** Distribution of risk score (up) and ischemic status (down) of atherosclerotic patients, survival curve, time-dependent ROC curve of the risk model of the atherosclerotic patients in the high and low-risk groups in test cohort, respectively. **(D–F)** Distribution of risk score (up) and ischemic status (down) of atherosclerotic patients, survival curve, time-dependent ROC curve of the risk model of the atherosclerotic patients in the high and low-risk groups in the overall cohort, respectively. **(G–I)** Distribution of risk score (up) and ischemic status (down) of atherosclerotic patients, survival curve, time-dependent ROC curve of the risk model of the atherosclerotic patients in the high and low-risk groups in PBMCs cohort, respectively.

To further test the stability of the risk scores, the predictive value was validated in the training cohort, entire cohort, and PBMCs cohort of the GSE21545 dataset. The test cohort was classified into low- and high-risk groups, and the risk score distributions and expression patterns of seven genes are presented in Figure 9A; Supplementary Figure S6A, respectively. The KM survival analysis showed that patients with high-risk scores demonstrated a prominent poor prognosis (log-rank test, *p* < 0.003; HR = 11.453; Figure 9B). The ROC curve showed that risk scores in the test cohort exhibited a good predictive value considering 1-, 3-, and 5-year AUC, which were 0.72, 0.79, and 0.83, respectively. The proportion of the two clusters and age groups in the two risk groups did not significantly differ (Supplementary Figures S6B, S6D), whereas ischemic events were more common in the high-risk group than the low-risk group (40.0% vs. 4.0%; Chi-square test, *p* = 0.002; Supplementary Figure S6C). The Sankey diagram depicted the relationships among the different groups (Supplementary Figure S6D). We further tested the predictive value of risk scores in the overall cohort and divided them into high- and low-risk groups according to the distributions of risk scores (Figure 9D). KM analysis indicated that patients with a high-risk score had worse prognosis than patients with a low-risk score (Figure 9E). Values of 1-, 3-, and 5-year AUC for ROC analysis were 0.84, 0.84, and 0.86, respectively, in the entire cohort (Figure 9F). Furthermore, we observed that the proportion of cluster 1, the occurrence of an ischemic event, and older patients in the high-risk group was significantly higher than those in the low-risk group (Supplementary Figures S7A–S7D). To further investigate whether the risk model is applicable to other tissues, we recruited the PBMCs cohort in the GSE21545 dataset and observed a significant difference in the prognosis of patients in the high- and low-risk groups (Figures 9G,H). Furthermore, the 1-, 3-, and 5-year AUC values for assessing the predictive accuracy of risk scores were acceptable (Figure 9I; 1-year AUC: 0.63; 3-year AUC: 0.60; 5-year AUC: 0.69). Moreover, the high-risk group had a significantly higher proportion of ischemic events, while age group did not significantly differ (Supplementary Figures S7E–S7G).

In addition, a subgroup analysis in the entire plaque cohort indicated that the risk scores in older patients are significantly greater than those in young patients (Supplementary Figure S8A). KM analysis showed that the risk model still exhibited potent predictive performance, and both old and young patients with lower risk scores had better prognosis (Supplementary Figures S8B, S8C; log-rank test, *p* < 0.001).

### Construction of a nomogram

**FIGURE 10:**
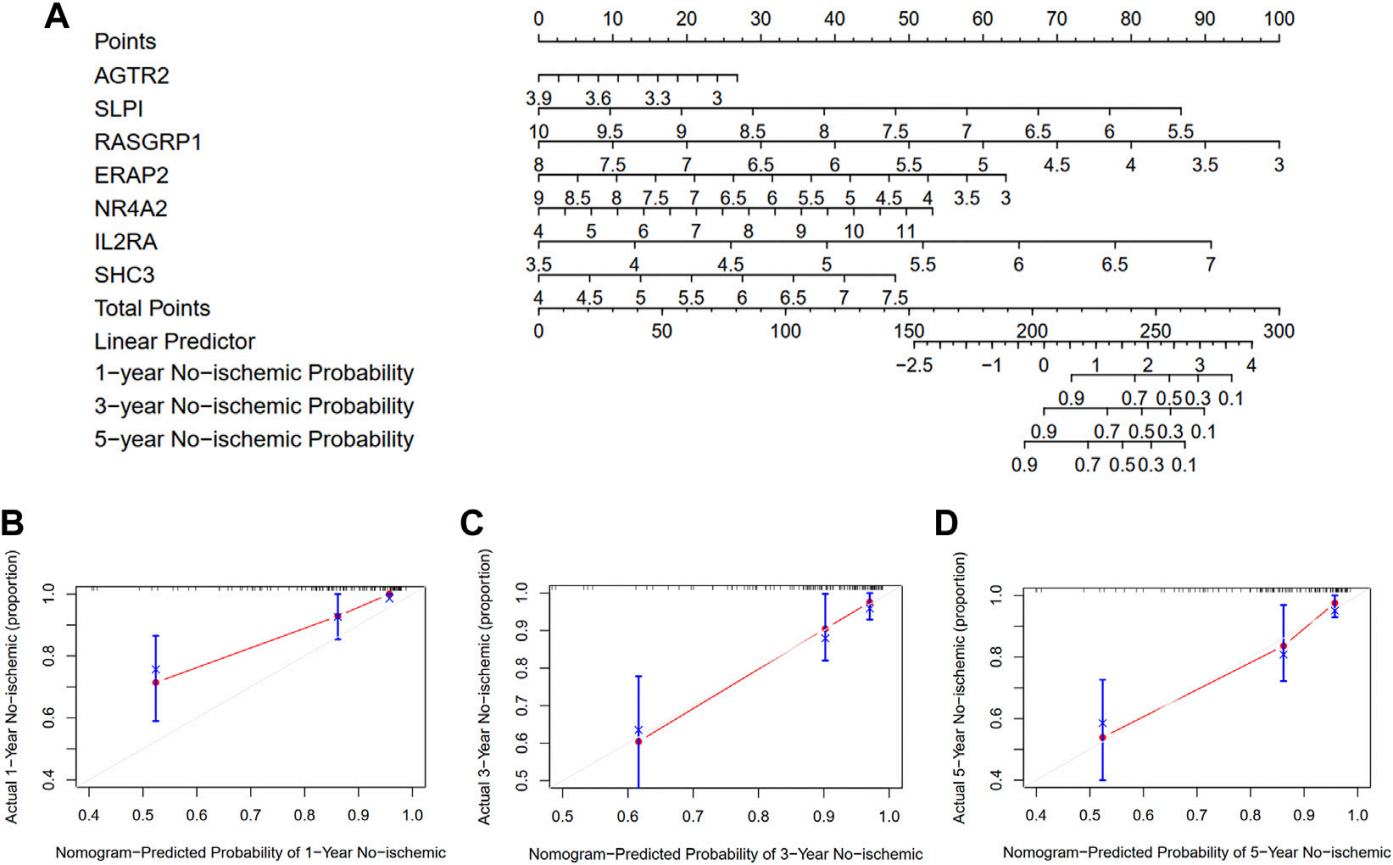
Development and evaluation of the nomogram to predict the ischemic event for patients with atherosclerosis undergoing CEA. **(A)** A combination of seven genes’ expressions was used to construct a nomogram for predicting the 1-, 3-, and 5-year event-free probability. **(B–D)** Calibration curves demonstrate that the nomogram-predicted event-free probabilities correspond closely to the observed probabilities for 1-, 3-, and 5-year in patients with atherosclerotic plaque, respectively.

To quantify the risk assessment of individual patients with atherosclerotic plaque and enhance the clinical applicability, we constructed a nomogram with the seven genes to predict the probability of 1-, 3-, and 5-year ischemic event-free probability (Figure 10A). Calibration curves assessing the performance of the nomogram demonstrated a satisfactory match between the actual and nomogram-predicted 1-, 3-, and 5-year ischemic event-free probabilities (45° line, Figures 10B–D).

## Discussion

Narrowing of the internal carotid artery in atherosclerosis is the underlying cause of IS in 8%–15% cases (Bonati et al., 2022). Underneath a monolayer of endothelial cells (ECs) that line the interior vessel wall, a lifetime long accumulation and transformation of lipids, inflammatory cells, smooth muscle cells, and necrotic cell debris in the intimal space shapes the atherosclerotic lesions. The rupture of the atherosclerotic plaque with subsequent embolism of a locally formed thrombus or plaque debris is the most important factor causing stroke or TIA. Previous studies ascribed this process to inflammation in the plaque (Ross, 1999). IRGs involved in the development, progression, and rupture of plaque through bioinformatics analysis have been widely studied (Li et al., 2022; Yang et al., 2022). Moreover, an immune-related prognostic signature to predict the overall survival in various cancers has been established recently (Dai et al., 2021). CEA has an established role in the treatment of patients with symptomatic or asymptomatic significant carotid artery stenosis. Prognosis after CEA is mainly determined by the occurrence of major adverse cardiovascular events, encompassing myocardial infarction, stroke, and cardiovascular death. How IRGs help predict ischemic events in patients after CEA is still unclear from the perspective of prognosis. In this study, we discussed the possibility that IRGs will elucidate the prognosis of patients with atherosclerosis and improve the risk prediction of ischemic events based on the gene expression data from CEA samples.

First, compared with the normal arterial intima, the atherosclerotic plaque exhibited enrichment of immune-related pathways and apoptosis process in our GSEA. Then, we identified the DEIRGs between plaque and control samples, among which nine prognostic IRGs were further screened using univariate Cox regression. On the basis of the nine prognostic IRGs, the carotid plaque samples were classified into two different immune-related clusters. The biological functions and immune characteristics between the two clusters were further elucidated. Moreover, the immune index of each sample was calculated by PCA, and the ARGs in plaque were detected using Pearson’s correlation method and the WGCNA algorithm. Next, using LASSO and multivariate Cox regression, a prognostic model with seven hub genes (IL2RA, NR4A2, DES, ERAP2, SLPI, RASGRP1, and AGTR2) was constructed and confirmed for patients with atherosclerotic plaque. Furthermore, we established a nomogram based on the seven-gene signature that can be used in clinical practice.

Our GSEA results showed that the inflammatory response, IL6/JAK/STAT3 signaling, complement activation, and interferon-gamma response were upregulated in atherosclerotic plaque, consistent with the literature (Fernandez and Giannarelli, 2022). Interleukin-6 (IL-6) is a macrophage secretory product that is abundantly expressed in atherosclerotic plaques (Tyrrell and Goldstein, 2021). During early Angiotensin II-induced atherosclerosis, IL-6, produced by activated macrophages and fibroblasts in the aortic adventitia, can induce the JAK-STAT3 pathway (Recinos et al., 2007). Selective inhibition of the JAK-STAT3 signaling pathway can repeal ATP-binding cassette transporter A1 (ABCA1)-mediated cholesterol efflux stimulated by IL-6 (Frisdal et al., 2011). On the other hand, IL-6 can attenuate the macrophage pro-inflammatory phenotype by preventing the accumulation of cytotoxic-free cholesterol and reduce both foam cell formation, the accumulation of apoptotic bodies, and intraplaque inflammation in atherosclerotic plaque. Apart from IL-6, interferon (IFN)-γ derived from T cells is expressed at high levels in atherosclerotic lesions. However, whether the roles of IFN-γ are pro- or antiatherogenic is still controversial. IFN-γ can play a pro-atherogenic role by recruiting immune cells to the atherosclerotic lesion (Buono et al., 2003), increasing the production of chemokines (Valente et al., 1998), and activating immune cells (Schroder et al., 2004). Despite the various pro-atherogenic functions, IFN-γ can also protect against atherosclerosis. For example, IFN-γ can inhibit macrophage-mediated LDL oxidation (Fong et al., 1994) and decrease the expression of very low density lipoprotein receptor and foam cell formation by remnant lipoproteins (Kosaka et al., 2001).

Considering the complexity of the immune microenvironment in plaques, we further explored the relationships between IRGs and immune characteristics with the prognosis of patients after CEA. Combined with DEGs in the GSE97210 dataset and univariate Cox regression analysis in GSE21545 plaque samples, nine IRGs with prognostic ability were screened out, and the plaque samples were grouped into two clusters with different prognosis based on the expression patterns of the nine IRGs. KM survival analysis showed that cluster 1 had a worse prognosis than cluster 2. Moreover, cluster 1 had a higher proportion of patients with occurrences of ischemic events during follow-up. Unexpectedly, in our immune infiltration analysis through xCell and ssGSEA methods, we found that cluster 2 with a better prognosis had a higher immune score and stronger inflammatory responses and immune cell infiltration. In our next risk-stratification analysis, we also observed that the low-risk group had higher immune characteristics than the high-risk group. This seemed contrary to the dominant perception: abundant infiltration of immune cells is associated with poorer clinical outcomes in patients with atherosclerosis. For example, a recent study showed that carotid plaque inflammation, identified by 18F-fluorodeoxyglucose (18FDG)-PET, improved the identification of 5-year recurrent ipsilateral IS. Moreover, anti-inflammatory medications such as colchicine and canakinumab can reduce recurrent vascular events including stroke, as reported in randomized trials of patients with coronary disease (Ridker et al., 2017; Tardif et al., 2019). The abnormal results can be explained in many ways. First, not all immunotherapies are atheroprotective. The negative results of the CIRT trial (Ridker et al., 2019) showed that low-dose methotrexate in patients with previous myocardial infarction or multivessel CAD did not result in fewer cardiovascular events than placebo. Moreover, in the COPS trial (Tong et al., 2020) in patients with acute coronary syndrome, the addition of colchicine to standard medical therapy resulted in higher mortality and did not significantly affect cardiovascular outcomes at 12 months. In an animal experiment, cytokine therapy with IL-2/anti-IL-2 monoclonal antibody complexes could attenuate the development and progression of atherosclerosis by increasing CD4^+^CD25+Foxp3+ regulatory T cells in apolipoprotein E-deficient mice (Dinh et al., 2012). Ongoing LILACS (Zhao et al., 2018) clinical trials have adopted new strategies that utilize antiatherosclerotic functions of the immune system, such as using low-dose IL-2 to promote the polarization of antiatherosclerotic regulatory T (Treg) cells. Second, the immune composition and subsets of human plaques are diverse and associated with clinical cardiovascular events. A recent study depicted a single-cell immune landscape of human atherosclerotic plaques and revealed that although in symptomatic patients, both plaque CD4^+^ and CD8^+^ T cells presented gene expression signatures largely consistent with differentiation and exhaustion, T cells were mostly activated in plaques of asymptomatic patients. Similar to plaque T cells, macrophages of asymptomatic patients were activated and exhibited a pro-inflammatory role, while macrophages from plaques of patients with recent cardiovascular events displayed reparative functions. Moreover, compared to symptomatic patients, plaque macrophages of asymptomatic patients expressed higher levels of IL1B and activated IL-1 signaling, which was targeted in the CANTOS trial to reduce the cardiovascular risk (Ridker et al., 2017). Therefore, despite the higher abundance of immune cell infiltration in cluster 2, the proportions of these complicated immunocyte subsets are still unclear in the present study. Third, the high expression levels of immune checkpoint proteins may serve as an atheroprotective factor. An increased incidence of atherosclerotic cardiovascular events is seen after the initiation of treatment with immune checkpoint inhibitors, potentially because of the accelerated progression of atherosclerosis (Drobni et al., 2020). In our study, we found that CD274, CD80, and CD47 were more pronounced in cluster 2 with a better prognosis than in cluster 1, but PDCD1 was down-regulated. Moreover, in our subsequent risk-stratification analysis, we also observed that the expression levels of immune checkpoint genes in the low-risk group were higher than those in the high-risk group. Finally, the characteristics of atherosclerotic plaques are also associated with the risk of stroke. A recent meta-analysis (Kamtchum-Tatuene et al., 2020) indicated that the incidence of ipsilateral ischemic cerebrovascular events was higher in patients with high-risk plaques (neovascularization, echolucency, and lipid-rich necrotic core) than in patients without high-risk plaques. However, due to the loss of clinical and pathological information, the characteristics of atherosclerotic plaques were unavailable in our work, which may influence the final results. These abovementioned points explain why cluster 2 and the low-risk group in our subsequent analysis with better prognosis had a higher immuneresponse. Therefore, our finding does not contrast the previous classical concept but enhances our understanding of human atherosclerotic plaque.

In our risk-stratification analysis, seven hub genes (IL2RA, NR4A2, DES, ERAP2, SLPI, RASGRP1, and AGTR2) were screened out using the LASSO method and used to calculate the risk score in each sample by using multivariate Cox regression. Survival analysis revealed that in the training and verification cohorts, the established risk model exhibited potent predictive performance for the ischemic events in patients with atherosclerosis. Angiotensin II type 2 receptor (AGTR2) exerts antiproliferative, antifibrotic, and proapoptotic effects in the vasculature. The loss of AGTR2 during the evolution of atherosclerotic lesions enhanced the cellularity of atherosclerotic lesions by decreasing the percent positive area of macrophages, smooth muscles, lipids, and collagen (Sales et al., 2005). However, one study suggested that AGTR2 blockers could prevent diabetes-associated atherosclerosis (Koïtka et al., 2010). In our study, we found AGTR2 as a protective gene in the univariate Cox regression. IL2RA encodes IL-2 receptor subunit α, which regulates lymphocyte activation and plays an important role in atherosclerosis. Through genome-wide association analysis, Lange et al. found that soluble IL-2Rα is positively associated with clinical cardiovascular events, and 52 single-nucleotide polymorphisms in the chromosome 10p15-p14 region containing IL2RA reached genome-wide significance (Durda et al., 2015). In a multi-ethnic study of atherosclerosis, soluble IL-2Rα was found to be associated with a higher risk of incident heart failure (Bakhshi et al., 2020). In our analysis, we observed that IL2R acts as a risk factor for the poor prognosis after CEA. Secretory leukocyte protease inhibitor (SLPI), a serine protease inhibitor, inhibits proteases, exerts antimicrobial activity, and inhibits nuclear factor-kappa B (NF-κB)-mediated inflammatory gene transcription (Nugteren and Samsom, 2021). SLPI can attenuate NF-κB-dependent inflammatory responses of human ECs and macrophages to atherogenic stimuli through the reduction of endothelial IL-8 release (Henriksen et al., 2004). The inflammatory suppression role of SLPI may confer protection against atherosclerosis by neutralizing the effect of inflammatory factors such as TNF-α or by blocking the activation of NF-κB. The role of SLPI in the prognosis of atherosclerosis was demonstrated. The NR4A subfamilies belong to the nuclear hormone receptor superfamily and are early response genes that encode proteins in response to various stressors. NR4A2 is one of the members of the NR4A family that also comprises NR4A1 and NR4A3. In our univariate Cox regression, NR4A2 and NR4A3 were identified owing to their prognostic ability. In contrast to the antagonistic relationship between SLPI and NF-κB, the transcriptional induction of NR4A1, NR4A2, and NR4A3 expression can be triggered through the NF-κB signaling pathway by treating macrophages with lipopolysaccharide (LPS), cytokines, or oxidized lipids (Pei et al., 2005). Desmin, encoded by DES, is the primary intermediate filament of cardiac, skeletal, and smooth muscles. Early study found that the intermediate filament inhibitor ivabradine indirectly reduced the expression of anti-arteriogenic cytokines CXCL10 and CXCL11 and desmin in ApoE(−/−) hindlimb tissue (Schirmer et al., 2012). The expression of desmin was also reported to be directly inhibited by elevated miR-338-3p, thus promoting the contractile-to-synthetic vascular smooth muscle cell phenotypic transition (Yan et al., 2021). We found that DES gene is a negative marker for the prognosis of atherosclerosis. To our knowledge, there is no direct correlation of ERAP2 and RASGRP1 with atherosclerosis, which may provide future experimental directions to elucidate the pathogenetic and prognostic mechanisms of atherosclerosis.

In general, this work stratified two plaque clusters with significantly different prognoses based on IRGs. Moreover, a prognostic model with seven hub IRGs was established and validated. We also found that the low-risk group had a significantly better prognosis than the high-risk group. Finally, a nomogram based on the seven genes was constructed, which might have future implications in clinical care by helping identify high-risk subjects who should be provided tailored treatment to more effectively prevent ischemic events. The findings of this study could help optimize the classification of atherosclerosis patients and also provide a new perspective and direction for future research on molecular targeted therapy.

Nevertheless, there are some limitations to our study. First, although the GSE21545 dataset provided follow-up information, more clinical (such as cardiovascular risk factors or drug treatment) and pathological data for each patient were unavailable to us. For example, plaque characteristics can help identify a high-risk patient more precisely. Second, we performed intracohort cross-validation of our risk score. Although the risk model showed a relatively great accuracy for the prognosis in different tissues (plaque and PBMCs), an independent cohort validation or a prospective cohort could provide more robust evidence for assessing its clinical utility. Finally, in this study, all the results were acquired by bioinformatics analysis, and we have not conducted any *in vivo* or *in vitro* experiment to verify the different expression levels in our study.

## Conclusion

In this study, we identified two molecular subgroups based on their expression patterns of IRGs through NMF clustering. The two molecular subgroups showed significantly different prognosis and immune status. A prognostic model based on seven IRGs (IL2RA, NR4A2, DES, ERAP2, SLPI, RASGRP1, and AGTR2) was developed, and its prediction efficiency was well verified. This study provides proof of concept that use of a signature based on IRGs from the atherosclerotic plaque could provide a novel insight that can aid the prediction of ischemic events and the study of molecular mechanisms and targeted therapies for atherosclerosis.

## Data Availability

The datasets presented in this study can be found in online repositories. The names of the repository/repositories and accession numbers can be found in the article/Supplementary Material.
