# Corrigendum: Construction of an immune-related signature for predicting the ischemic events in patients undergoing carotid endarterectomy

**DOI:** 10.3389/fgene.2022.1104846

**Published:** 2023-01-06

**Authors:** Shifu Li, Qian Zhang, Ling Weng, Jian Li

**Affiliations:** ^1^ Department of Neurosurgery, Xiangya Hospital, Central South University, Changsha, China; ^2^ National Clinical Research Center for Geriatric Disorders, Central South University, Changsha, China; ^3^ Department of Neurology, Xiangya Hospital, Central South University, Changsha, China; ^4^ Hydrocephalus Center, Xiangya Hospital, Central South University, Changsha, China

**Keywords:** atherosclerosis, immune-related genes, immune infiltration, ischemic events, molecular subtypes

In the published article, there was an error.

A correction has been made to **[Discussion]**, [Paragraph 3]. This sentence previously stated:

“IFN-γ can inhibit macrophage-mediated LDL oxidation (Fong et al., 1994) and reduce the uptake of oxidized LDL that contributes to atherosclerotic plaque formation and progression (Kosaka et al., 2001).”

The corrected sentence appears below:

“IFN-γ can inhibit macrophage-mediated LDL oxidation (Fong et al., 1994) and decrease the expression of very low density lipoprotein receptor and foam cell formation by remnant lipoproteins (Kosaka et al., 2001).”

The authors apologize for this error and state that this does not change the scientific conclusions of the article in any way. The original article has been updated.

