# Supplementary material for: Construction of an immune-related signature for predicting the ischemic events in patients undergoing carotid endarterectomy
Source: Front Genet. 2022 Oct 10;13:1014264. doi: 10.3389/fgene.2022.1014264 (PMC9592116; doi:10.3389/fgene.2022.1014264)
Supplement: Supplementary file 1 [file Table1.DOCX]

Table 1. Detailed information of GSE21545 datasets.

| Samples |  | Plaque (126) |  |  | PBMCs (97) |
| --- | --- | --- | --- | --- | --- |
|  |  | Training Cohort (76) | Test Cohort (50) | Overall Cohort (126) |  |
| Status | Ischemic | 14 (18.42%) | 11 (22.00%) | 25 (19.84%) | 21 (21.65%) |
|  | No-Ischemic | 62 (81.58%) | 39 (78.00%) | 101 (80.15%) | 76 (78.35%) |
| Age | <=65 | 21 (27.63%) | 14 (28.57%) | 35 (28.00%) | 27 (71.88%) |
|  | >65 | 55 (72.37%) | 35 (71.43%) | 90 (72.00%) | 69 (28.12% |

PBMCs, peripheral blood mononuclear cells.
