# Supplementary material for: Construction of an immune-related signature for predicting the ischemic events in patients undergoing carotid endarterectomy
Source: Front Genet. 2022 Oct 10;13:1014264. doi: 10.3389/fgene.2022.1014264 (PMC9592116; doi:10.3389/fgene.2022.1014264)
Supplement: Supplementary file 2 [file Table2.DOCX]

Table 2. Gene descriptions and univariate Cox regression of nine prognostic immune-related genes.

| Gene name | Gene description | Chromosome | Gene type | HR (95%CI) | P value |
| --- | --- | --- | --- | --- | --- |
| SLPI | secretory leukocyte peptidase inhibitor | 20 | protein_coding | 0.58 ( 0.35 - 0.98 ) | 0.0399 |
| SHC3 | SHC adaptor protein 3 | 9 | protein_coding | 2.4 ( 1.1 - 4.9 ) | 0.0219 |
| RASGRP1 | RAS guanyl releasing protein 1 | 15 | protein_coding | 0.58 ( 0.37 - 0.93 ) | 0.0245 |
| NR4A3 | nuclear receptor subfamily 4 group A member 3 | 9 | protein_coding | 1.5 ( 1.1 - 2.1 ) | 0.0122 |
| NR4A2 | nuclear receptor subfamily 4 group A member 2 | 2 | protein_coding | 1.3 ( 1 - 1.8 ) | 0.0459 |
| IL2RA | interleukin 2 receptor subunit alpha | 10 | protein_coding | 2.4 ( 1 - 5.6 ) | 0.0388 |
| ERAP2 | endoplasmic reticulum aminopeptidase 2 | 5 | protein_coding | 0.73 ( 0.57 - 0.95 ) | 0.0175 |
| DES | desmin | 2 | protein_coding | 1.7 ( 1 - 2.8 ) | 0.04 |
| AGTR2 | angiotensin II receptor type 2 | X | protein_coding | 0.06 ( 0.0051 - 0.72 ) | 0.0266 |

CI, confidence interval.
