# Supplementary material for: Construction of an immune-related signature for predicting the ischemic events in patients undergoing carotid endarterectomy
Source: Front Genet. 2022 Oct 10;13:1014264. doi: 10.3389/fgene.2022.1014264 (PMC9592116; doi:10.3389/fgene.2022.1014264)
Supplement: Supplementary file 4 [file Table3.DOCX]

Supplementary Pictures


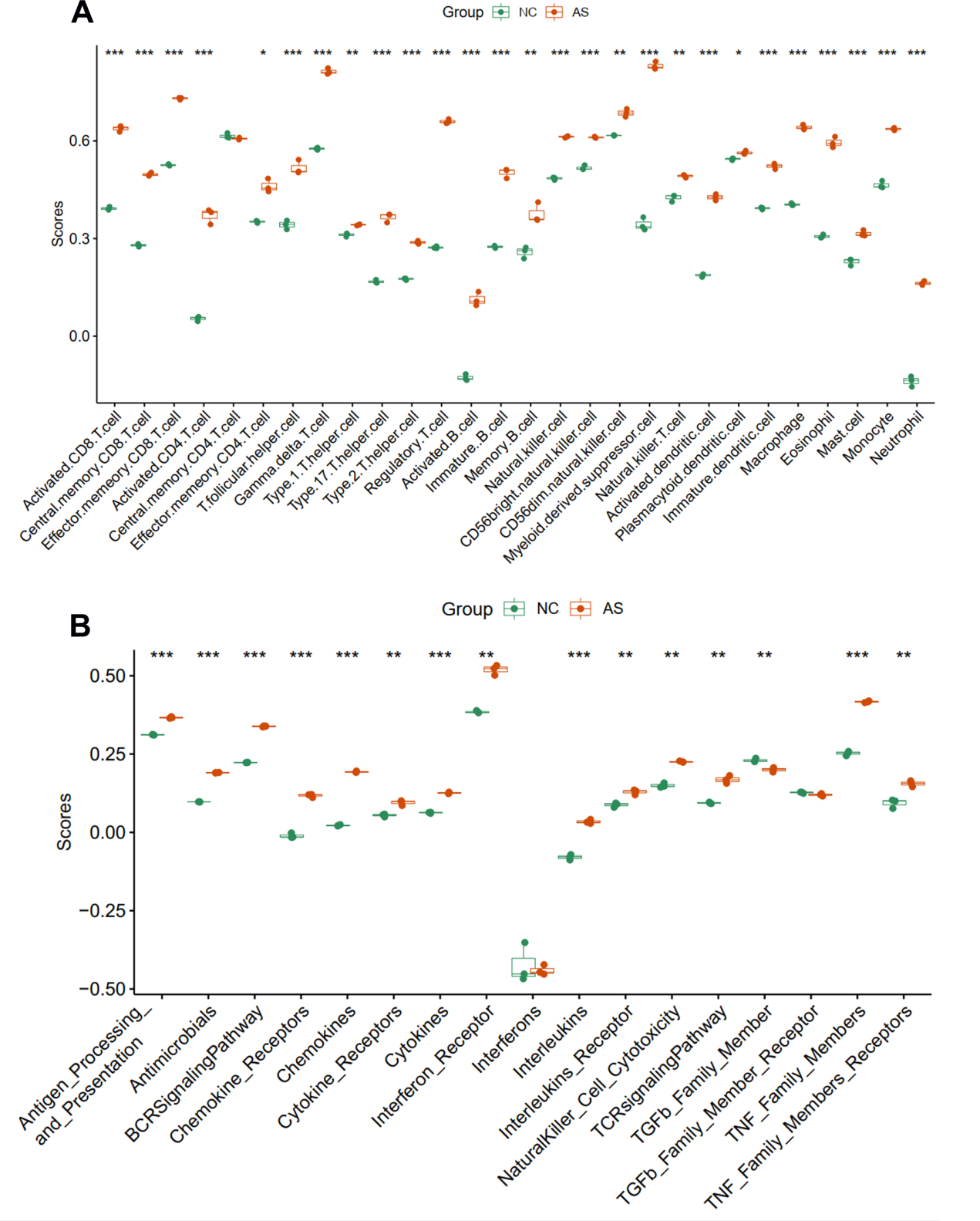


Figure S1. (A) The comparison of 28 immune cells between atherosclerotic plaque and controls with ssGSEA analysis. (B) The comparison of 17 immune responses between atherosclerotic plaque and controls with ssGSEA analysis. Significance level was denoted by *p‐value < .05, **p‐value < .01, ***p‐value < .001.


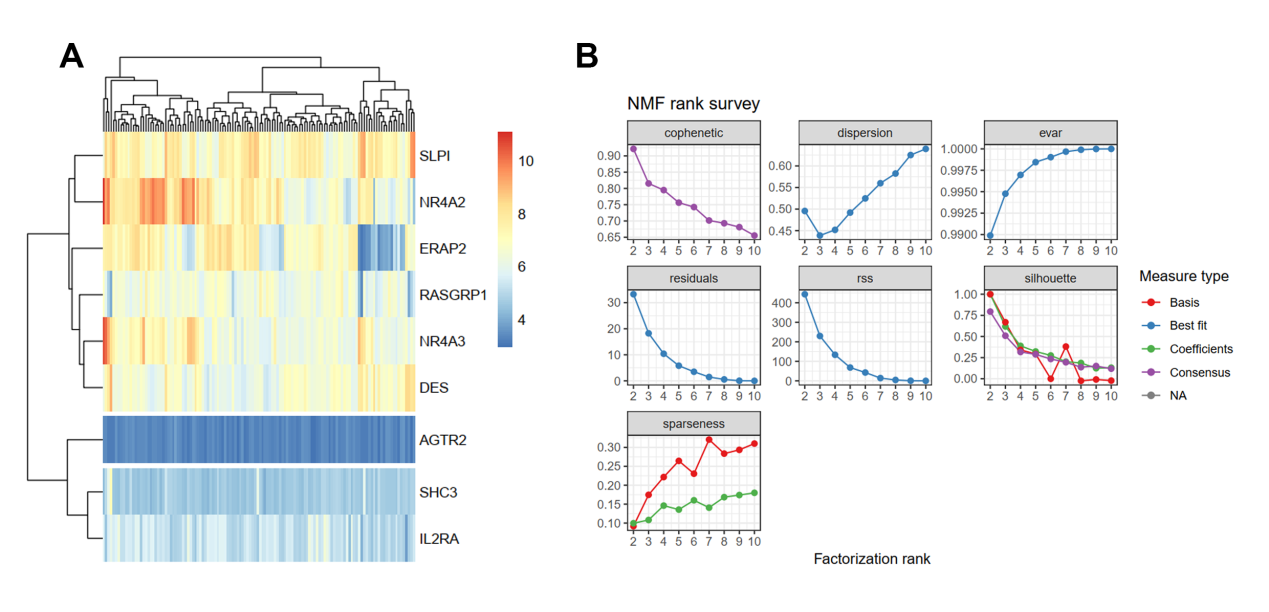


Figure S2. (A) Hierarchical clustering of the nine prognostic genes in GSE21545 dataset. (B) Estimation of the rank 2-10 with different NMF rank method.


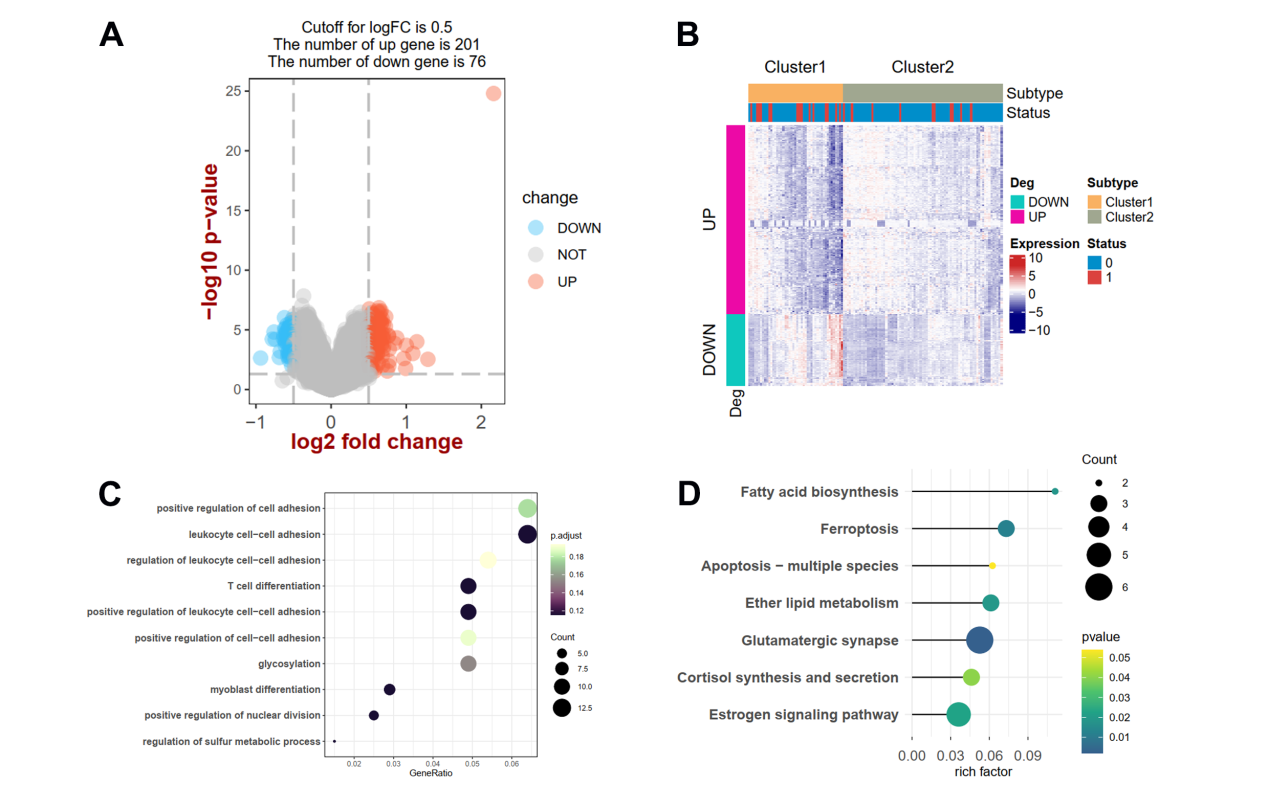


Figure S3. The differentially expressed genes and biological annotations between two clusters. (A) A volcano plot showing the DEGs between two clusters. (B) A heat map showing the espression patterns of DEGs. (C) Biological processes of DEGs in GO analysis. (D) The KEGG pathways of DEGs.


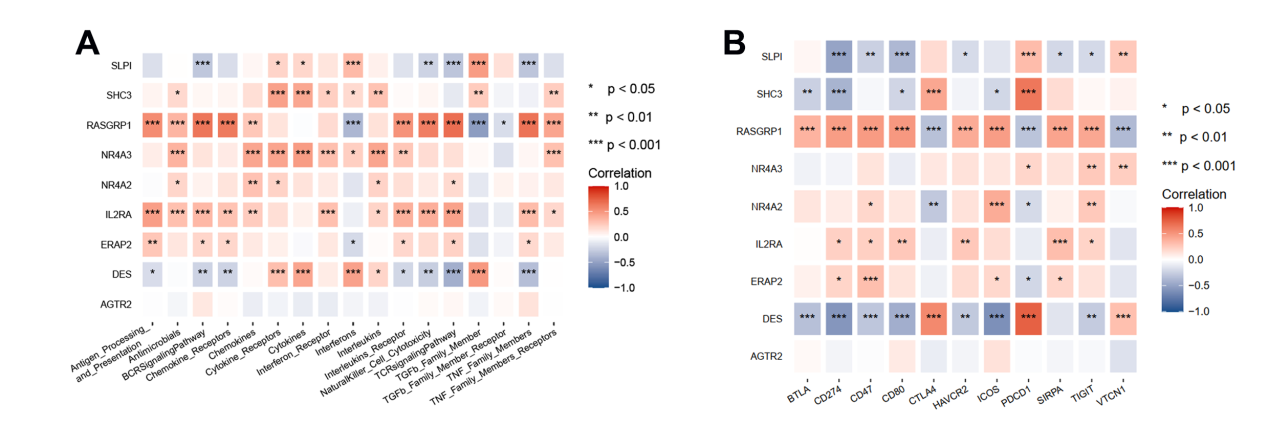


Figure S4. The relationships between nine prognostic IRGs with immune responses (A) and immune checkpoint genes (B).


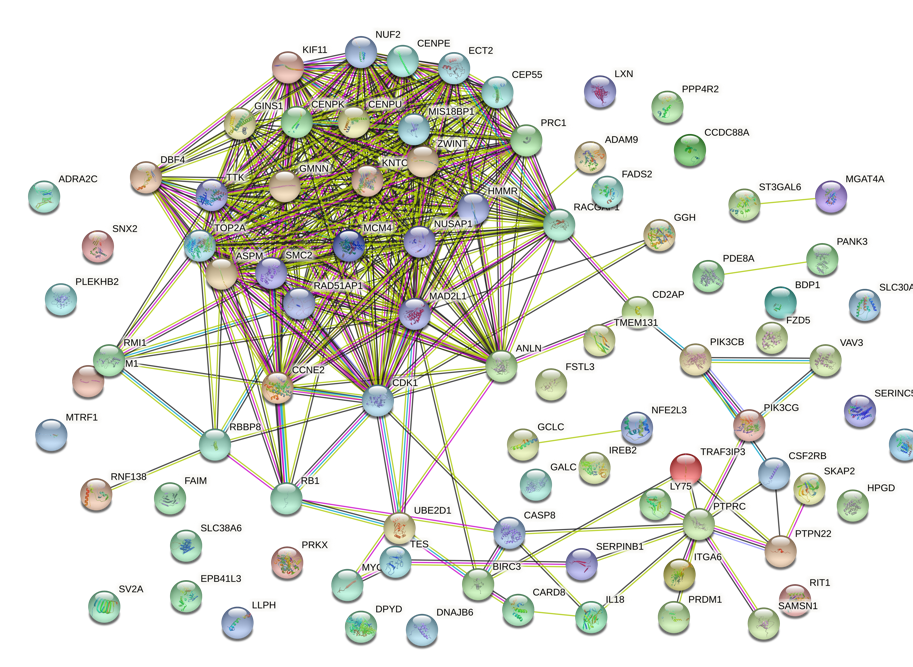


Figure S5. Protein-protein interaction network of 89 apoptosis-related genes.


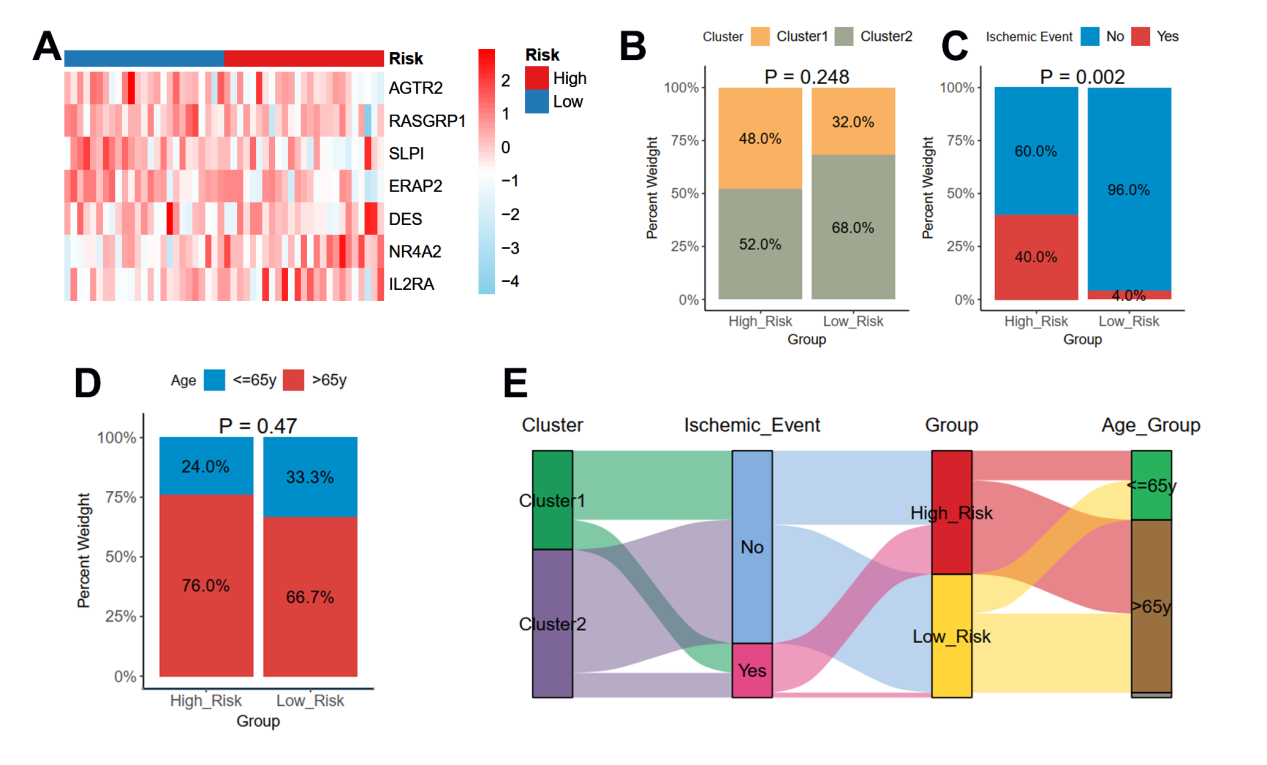


Figure S6. The clinical traits of high- and low-risk group in test cohort. (A) A heat map illustrating the expression patterns of the seven candidate genes in the two groups. The relative proportion of different clusters (B), ischemic event (C), and age group (D) in two risk group. (E) A Sankey diagram showing the distribution of different groups.


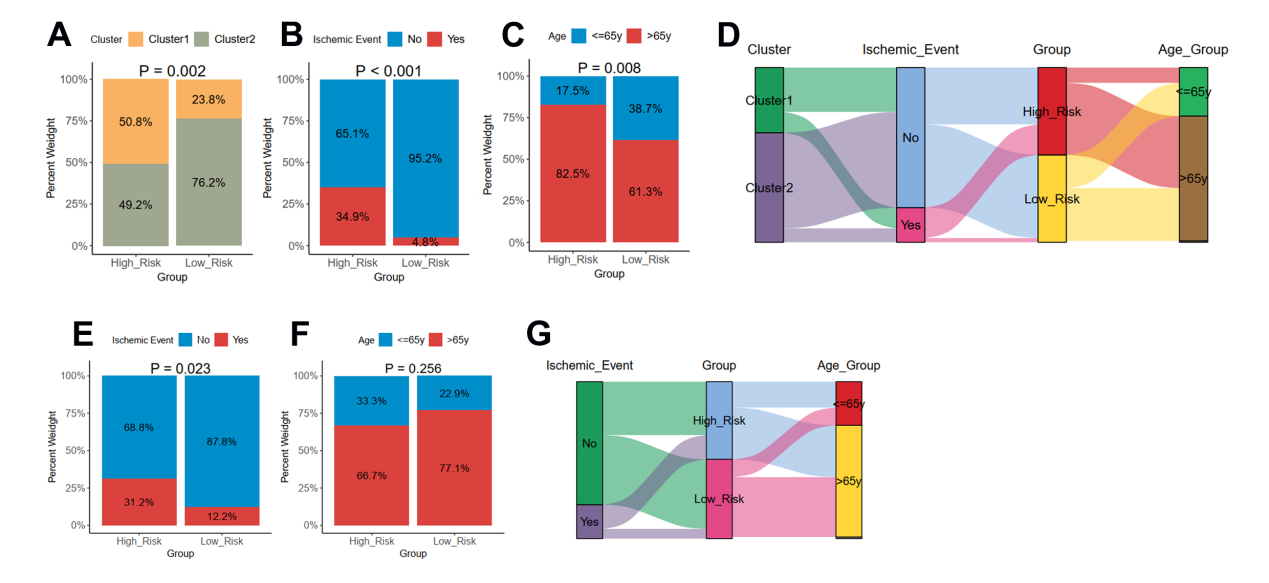


Figure S7. The clinical traits of high- and low-risk group in overall and PBMCs cohort. The relative proportion of different clusters (A), ischemic event (B), and age group (C) in two risk group in overall cohort. (D) A Sankey diagram showing the distribution of different groups in overall cohort. The relative proportion of ischemic event (E) and age group (F) in two risk group in PBMCs cohort. (G) A Sankey diagram showing the distribution of different groups in PBMCs cohort.


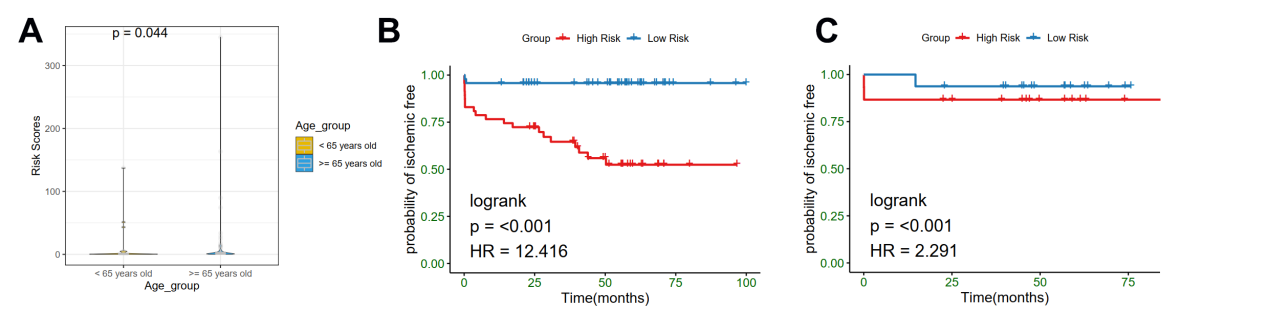


Figure S8. Subgroup analysis of the risk scores in different age. (A) Risk score was significantly higher in old patients than young patients. (B) Survive curve in patients >= 65 years old. (C) Survive curve in patients < 65 years old.
